# m6A-dependent mature miR-151-5p accelerates the malignant process of HNSCC by targeting LYPD3

**DOI:** 10.1186/s43556-024-00189-9

**Published:** 2024-07-16

**Authors:** Fei Huang, Yuan Ren, Yufei Hua, Ying Wang, Ruomeng Li, Ning Ji, Xin Zeng, Ding Bai, Qianming Chen, Xikun Zhou, Junjie Wu, Jing Li

**Affiliations:** 1 State Key Laboratory of Oral Diseases, National Clinical Research Center for Oral Diseases, Chinese Academy of Medical Sciences Research Unit of Oral Carcinogenesis and Management, West China Hospital of Stomatology, Sichuan University, Chengdu, 610041 Sichuan China; 2 State Key Laboratory of Biotherapy and Cancer Center, West China Hospital, Sichuan University and Collaborative Innovation Center for Biotherapy, Chengdu, 610041 China; 3 State Key Laboratory of Oral & Maxillofacial Reconstruction and Regeneration, Shaanxi Key Laboratory of Stomatology, Department of Orthodontics, School of Stomatology, National Clinical Research Center for Oral Diseases, The Fourth Military Medical University, Xi’an, 710032 China

**Keywords:** Head and neck cancer, miR-151-5p, Metastasis, N6-methyladenosine (m6A), hnRNP U

## Abstract

miRNA has emerged as a crucial regulator in various of pathological and physiological processes, yet its precise mechanism of action the detailed mechanism of their action in Head and neck squamous cell carcinoma (HNSCC) remains incompletely understood. This study sheds light on the role of mi-151-5p, revealing its significantly elevated expression in tumor cells, which notably enhances the invasion and migration of HNSCC cells. This effect is achieved through directly targeting LY6/PLAUR Domain Containing 3 (LYPD3) by miR-151-5p, involving complementary binding to the 3’-untranslated regions (3’-UTR) in the mRNA of LYPD3. Consequently, this interaction accelerates the metastasis of HNSCC. Notably, clinical observations indicate a correlation between high expression of miR-151-5p and low levels of LYPD3 in clinical settings are correlated with poor prognosis of HNSCC patients. Furthermore, our investigation demonstrates that glycosylation of LYPD3 modulates its subcellular localization and reinforces its role in suppressing HNSCC metastasis. Additionally, we uncover a potential regulatory mechanism involving the facilitation of miR-151-5p maturation and accumulation through N6-methyladenosine (m6A) modification. This process is orchestrated by methyltransferase-like 3 (METTL3) and mediated by a newly identified reader, heterogeneous nuclear ribonucleoprotein U (hnRNP U). These findings collectively underscore the significance of the METTL3/miR-151-5p/LYPD3 axis serves as a prominent driver in the malignant progression of HNSCC.

**Supplementary Information:**

The online version contains supplementary material available at 10.1186/s43556-024-00189-9.

## Introduction

Given the pervasive influence of miRNAs in regulating the spatiotemporal aspects of mRNA and protein synthesis, dysregulation in their expression holds significant potential to contribute to a spectrum of diseases, including the initiation and progression of malignancies [1]. Numerous studies have underscored the involvement of miRNAs in dictating the disease stage, histological type, and grading of Head and Neck Squamous Cell Carcinoma (HNSCC) [2–4], exhibiting both tumor-suppressive and oncogenic roles [5, 6]. Recently, attention has been drawn to a specific miRNA, miR-151, which has been extensively studied across various diseases. Notably, miR-151 has been implicated as a therapeutic target for systemic sclerosis [7], implicated in enhancing migration and invasion of hepatocellular carcinoma cells [8], and involved in mediating regulatory processes regulation in innate immunity and inflammation [9]. Despite being extensively investigated in over 130 studies, the precise mechanism underlying its actions in disease pathology remains elusive.

HNSCC stands as the sixth most prevalent cancer globally and ranks eighth among the leading causes of cancer-related deaths [1]. A notable proportion of HNSCC patients (20–40%) encounter local and/or regional disease recurrence accompanied by distant metastases [10]. Nonetheless, the intricate mechanism governing distant metastases in HNSCC remains incompletely understood. Recent insights have highlighted the contribution of miRNAs to the advancement of distant metastases in HNSCC, yet the specific mechanisms through which miR-151 participates in this process await elucidation.

The biogenesis of miRNAs entails a series of the processing steps involving nascent primary miRNA (pri-miRNA) transcripts, which undergo sequential cleavages. Initially these transcripts are cleaved by the enzyme DROSHA in the nucleus [11]. Notably, N6-methyladenosine (m6A) serves as a common internal RNA modification in eukaryotes, playing a pivotal role in facilitating the processing of pri-miRNA transcripts and thereby exerting downstream regulatory effects [12, 13]. However, the specific the precise maturation pathway of miR-151-5p in the context of HNSCC remains to be elucidated.

In this study, we provide compelling evidence implicating the m6A modification in the regulating of miR-151-5p and its consequential impact on the malignant evolution of HNSCC. We elucidate that miR-151-5p exerts its regulatory effect on LYPD3 expression through m6A modification. Specifically, the processing of pri-miR-151 is facilitated by the METTL3 enzymes alongside the recently identified hnRNP U reader protein, both crucial players in m6A modification. This process leads to the accumulation of mature miR-151-5p, consequently resulting in a pronounced reduction in LYPD3 expression. Remarkably, we uncover that glycosylation of LYPD3 promotes its subcellular localization, thereby impeding HNSCC metastasis. Consequently, the diminished expression of LYPD3 due to the accumulation of miR-151-5p accelerates the metastatic progression of HNSCC. Furthermore, our clinical data unveil a significant association between elevated levels of miR-151-5p and reduced levels of LYPD3 with unfavorable prognosis in HNSCC patients, underscoring their potential as valuable prognostic biomarkers and promising therapeutic targets. Collectively, our findings underscore the critical role of the METTL3/miR-151-5p/LYPD3 axis as a pivotal driver of the malignant progression of HNSCC.

## Results

### The oncogenic miR-151-5p is upregulated and correlates with invasion and migration in HNSCC cells

**Fig. 1:**
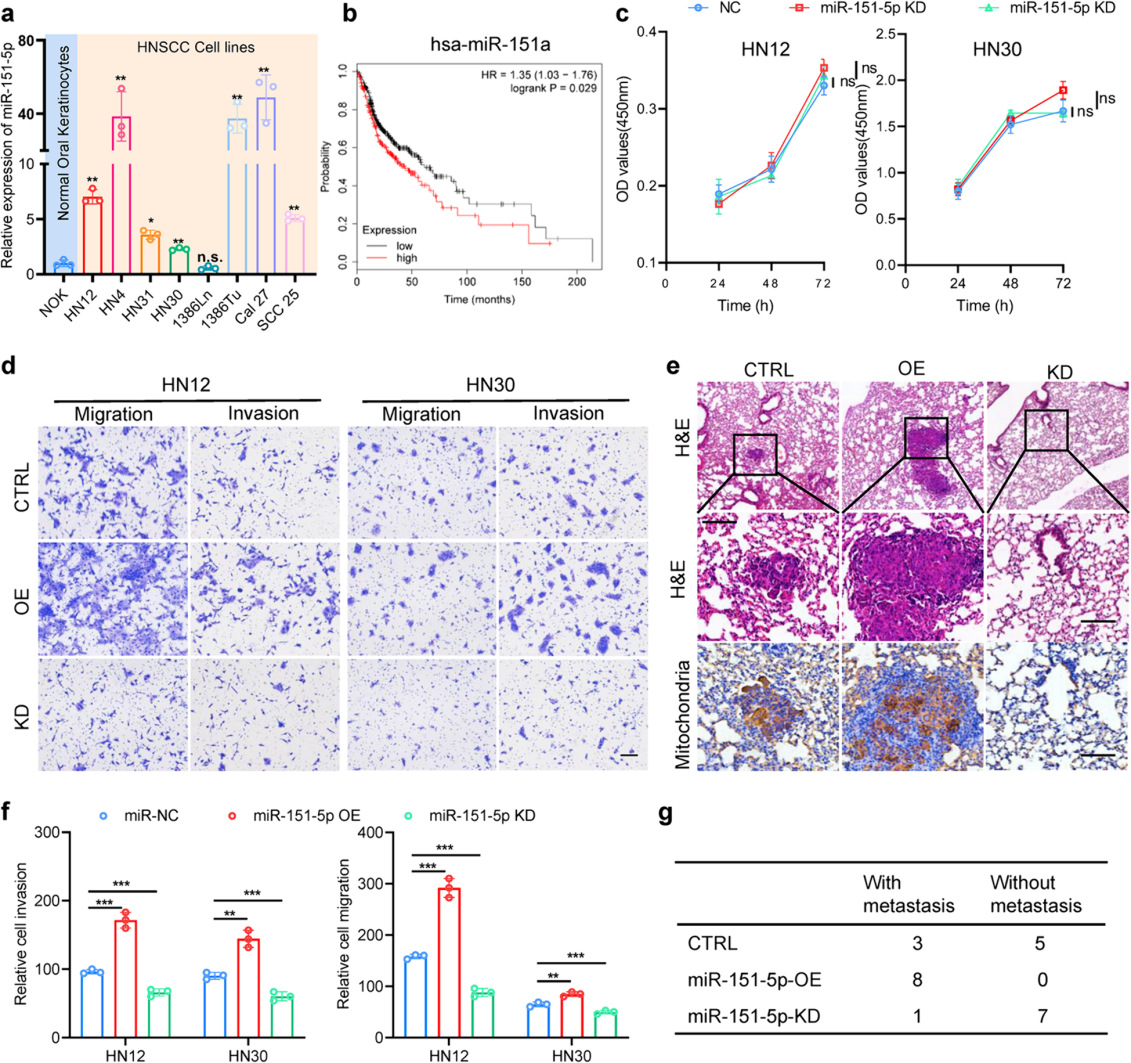
miR-151-5p is overexpressed in HNSCC cell lines and related to invasion and migration. **a** Relative expression levels of miR-151-5p detected by qRCP in NOK cells and various HNSCC cell lines. **b** Kaplan‒Meier plots from public databases depicting the shorter overall survival times of HNSCC patients with high miR-151-5p levels (≥ median) compared to those with low levels (< median). **c** CCK-8 assays were conducted to assess the effects of stable overexpression or knockdown of miR-151-5p on HN12 cell and HN30 cell proliferation in vitro. **d** The impact of stable overexpression or knockdown of miR-151-5p on the cell migration and invasion ability of HNSCC cells, with representative images of migration and invasion abilities shown in panels. **e** The impact of stable overexpression or knockdown of miR-151-5p on the migration of xenograft tumors derived from HN30 cells in nude mice, depicted through H&E slides and IHC with anti-human mitochondrial antibody in lung tissues from 8 mice in each group. **f** quantitative statistics of (**d**). **g** The number of lung metastasis stable overexpression or knockdown of miR-151-5p on the migration of xenograft tumors derived from HN30 cells in nude mice. Values are the mean ± S.D. from three or four independent experiments, and all statistical analyses in this figure are Student’s t test. *P < 0.05, **P < 0.01, ***P < 0.001 and NS, not significant as compared with the corresponding control

To determine precise contribution of miR-151-5p in HNSCC, we initially assessed its expression using q‒PCR across oral normal keratinocytes (NOK cells) and a panel of HNSCC cell lines. Our analysis revealed a widespread upregulation of miR-151-5p in most HNSCC cells, with the exception of 1386Ln, when compared to NOK cells (Fig. 1a). Additionally, Kaplan‒Meier plots sourced from public databases corroborated our findings, showing that elevated levels of miR-151a (pre-miR-151-5p) in HNSCC correlated with poorer prognosis compared to those with lower levels (Fig. 1b). To probe the functional implications of miR-151-5p in HNSCC, we conducted cell proliferation assays on two HNSCC cell lines, revealing no significant impact on cell proliferation (Fig. 1c). However, Transwell assays unveiled that knockdown (KD) of miR-151-5p reduced the migration and invasion capacities of HNSCC cells, whereas its overexpression (OE) enhanced these abilities (Fig. 1d, e). Moreover, validation through 3D multicellular tumor spheroid assays affirmed the heightened invasion potential of HNSCC cells upon stable overexpression miR-151-5p (Fig. S1b). Furthermore, these effects were recapitulated in mouse xenograft metastasis models utilizing HN30 cells, wherein miR-151-5p overexpression or knockdown resulted in lung metastasis percentages of 100% and 12%, respectively (Fig. 1e, g). Collectively, these findings suggest that miR-151-5p may play a pivotal role in accelerating the metastatic progression of HNSCC.

### miR-151-5p promotes invasion and migration of HNSCC cells by targeting LYPD3

**Fig. 2:**
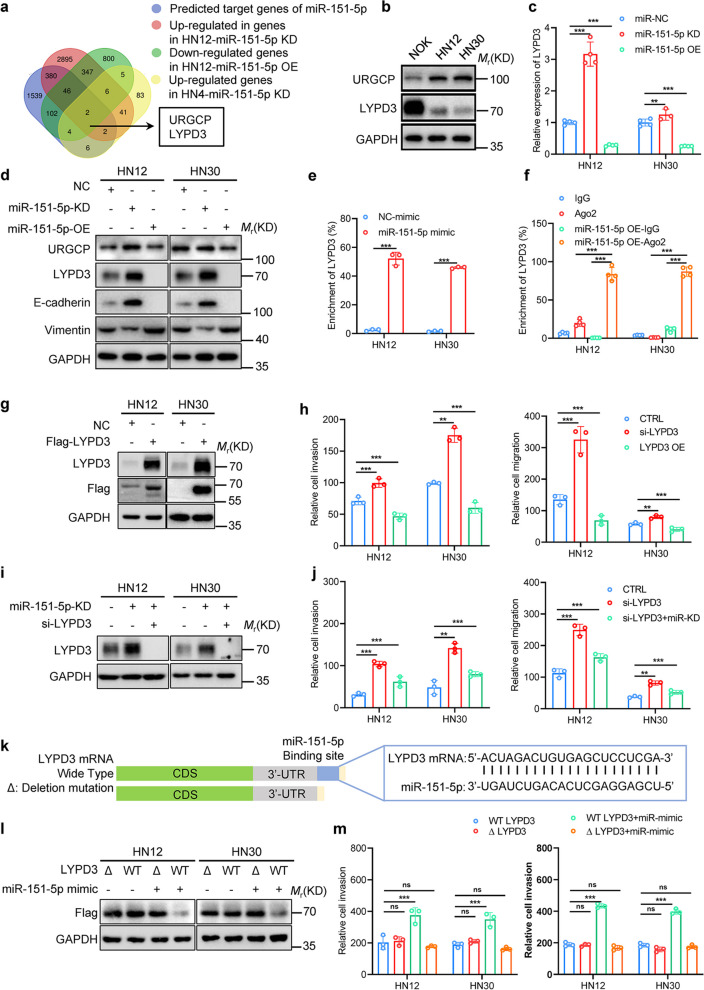
miR-151-5p inhibited the LYPD3 expression by binding to the LYPD3 mRNA 3’-UTR, resulting in the promotion of the invasion and migration of HNSCC cells. **a** The Venn diagram presents potential target genes of miR-151-5p, which intersect with genes predicted in the databases and show differential expression in HN12 cells transfected with miR-151-5p mimic and inhibitor and HN4 cells transfected with miR-151-5p inhibitor. **b** Western blot analysis indicates the differential expression of candidate miR-151-5p target genes in oral keratinocytes and HNSCC cells. **c** RNA levels of LYPD3 are impacted by stable overexpression or knockdown of miR-151-5p in HNSCC cells. **d** Western blot analysis indicates the differential expression of candidate genes of miR-151-5p and EMT protein in stable overexpression and knockdown of miR-151-5p HNSCC cells. **e** miRNA pull-down assays using biotin-labeled miR-151-5p mimic reveal the enrichment of LYPD3 RNA in HNSCC cells. **f** RNA immunoprecipitation assays with Ago2 demonstrate the enrichment of LYPD3 mRNA in HNSCC cells overexpressing miR-151-5p. **g** Western blot analysis confirmed LYPD3 overexpression with a FLAG tag in HNSCC cells. **h** The effects of LYPD3 knockdown and overexpression on HNSCC cell migration and invasion are shown, and quantitative statistics of migration and invasion abilities shown in panels. **i** Knockdown of LYPD3 expression by siRNA substantially rescued the expression of LYPD3 increased by miR-151-5p knockdown in HNSCC cells. **j** Knockdown of LYPD3 expression by transfecting with siRNA significantly reversed the effects of miR-151-5p knockdown on HNSCC cell migration and invasion, and quantitative statistics of migration and invasion abilities shown in panels. **k** Schematic representation of the mRNA structures of wild-type LYPD3 and the miR-151-5p binding site. Compared with LYPD3 wild type mRNA, Δ-LYPD3 mRNA lacks the miR-151-5p binding site in the 3′ untranslated region. **l** Transfected with miR-151-5p mimic didn’t affect the LYPD3 expression in mutant LYPD3 mRNA in HNSCC cells. **m** Mutation of LYPD3 mRNA significantly reversed the effects of miR-151-5p overexpression on HNSCC cell migration and invasion, and quantitative statistics of migration and invasion abilities shown in panels. All statistical analyses in this figure are Student’s t test unless specified. *P < 0.05, **P < 0.01, and ***P < 0.001 compared with the corresponding control

To elucidate potential target genes of miR-151-5p, we employed publicly available algorithms and analyzed RNA-seq data reflecting its expression alterations in HNSCC cells. Our analysis highlighted UGRCP and LYPD3 as targets (Fig. 2a). Subsequent evaluation of these candidates’ expression in normal oral keratinocytes and HNSCC cell lines revealed comparatively lower expression levels of LYPD3 in HNSCC cell lines, suggesting its candidacy as a tumor suppressor gene correlating with the oncogenic role of miR-151-5p (Fig. 2b). Further validation through qPCR and western blotting demonstrates that miR-151-5p overexpression downregulated both mRNA and protein expression of LYPD3, while miR-151-5p knockdown resulted in an elevated LYPD3 expression (Fig. 2c, d). Notably, UGRCP expression remained unchanged upon miR-151-5p modulation (Fig. 2d and Fig. S2a), emphasizing the specificity of LYPD3 as a target of miR-151-5p. Moreover, miRNA pull-down assays and Ago2 RIP assays revealed significant enrichment of LYPD3 mRNA in the miR-151-5p complex, further confirming its direct interaction with miR-151-5p (Fig. 2e, f and Fig. S2b, c).

Functional assessment through Transwell assays demonstrated that LYPD3 knockdown augmented the migration and invasion capabilities of HNSCC cells, whereas LYPD3 overexpression attenuated these abilities (Fig. 2g, h and Fig. S3a). Additionally, rescue experiments demonstrated that LYPD3 knockdown partially reversed the migration and invasion capabilities of HNSCC cells upon miR-151-5p knockdown (Fig. 2i, j and Fig. S3b). Remarkably, bioinformatic analysis unveiled a complete complementary binding site of within the 3’UTR of LYPD3 mRNA (Fig. 2k). Subsequent luciferase reporter assays corroborated this interaction, where the abolishment of the miRNA binding site in the mutant 3’-UTR of LYPD3 mRNA significantly mitigated miR-151-5p-mediated effects on HNSCC cell migration and invasion (Fig. 2l, m and Fig. S3c). Overall, these data underscore LYPD3 as the principal target gene of miR-151-5p, whose downregulation facilitates the migration and invasion of HNSCC cells.

### The glycosylation of LYPD3 modulates its subcellular localization and function

**Fig. 3:**
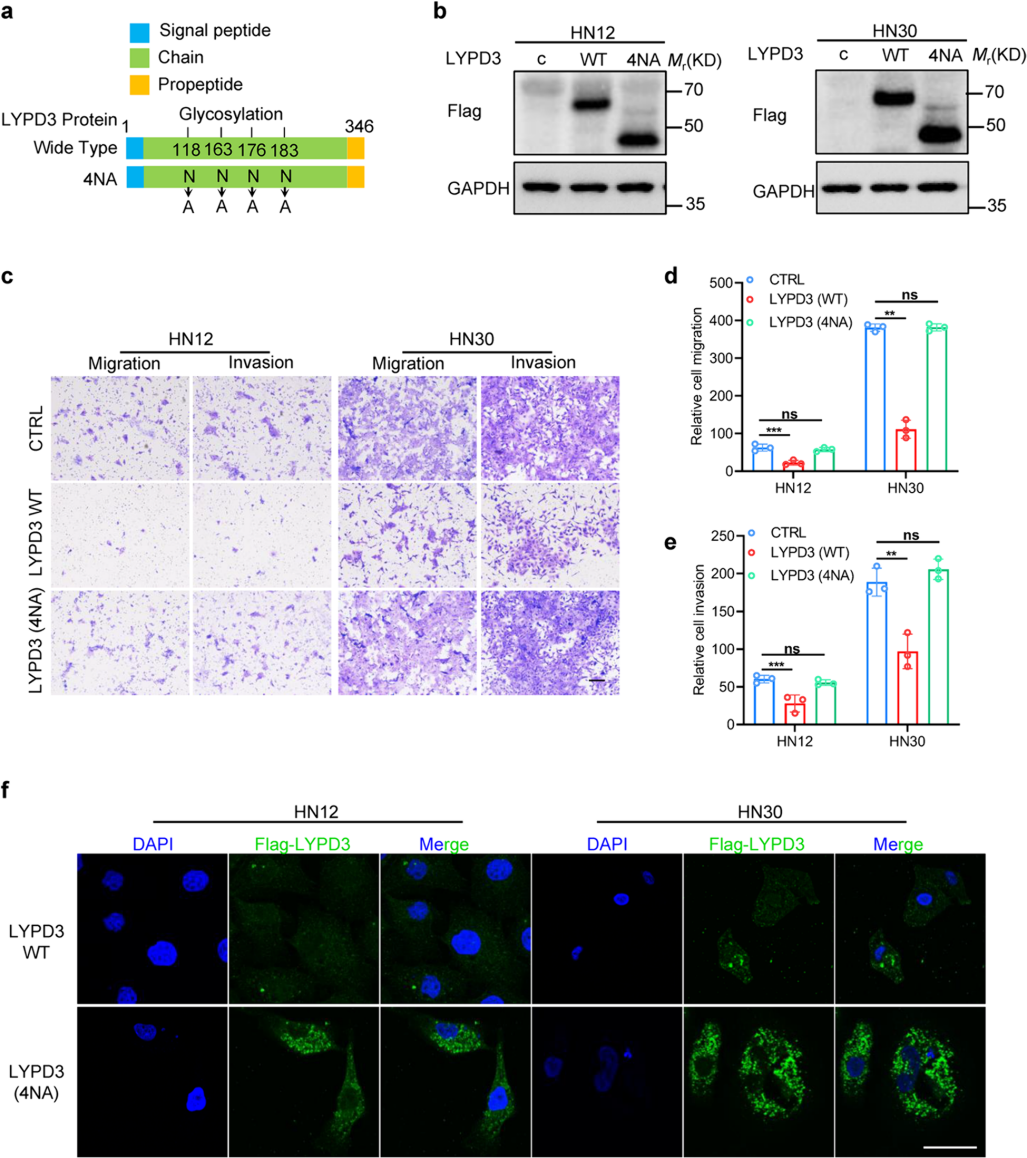
Glycosylation of LYPD3 mediates its subcellular localization and function. **a** The schematic depicts the protein structures and four glycosylation sites of LYPD3. The 118th, 163rd, 176th, and 183rd asparagine (N) residues were mutated into alanine (A) to generate LYPD3(4NA) versions. **b** Western blot analysis shows the expression and different molecular weight of stable expression of wild-type LYPD3 and LYPD3(4NA) whose 118th, 163rd, 176th, and 183rd asparagine (N) residues were mutated into alanine (A) in LYPD3 in HNSCC cells. **c**-**e** The effects of overexpression of wild-type LYPD3 and LYPD3(4NA) on the cell migration and invasion ability of HNSCC cells were evaluated through representative pictures of Transwell assays, indicating different cell migration and invasion abilities. Scale bars, 200 μm. **f** Subcellular localization of wild-type LYPD3 and LYPD3(4NA) in HNSCC cells was determined by immunofluorescence staining. Merge indicates combined immunofluorescence signals for Flag-tagged LYPD3 (green), and DAPI (blue) Scale bar: 10 μm. All statistical analyses in this figure are Student’s t test unless specified. *P < 0.05, **P < 0.01, and ***P < 0.001 compared with the corresponding control

While previous experiments have elucidated the pivotal role of miR-151-5p in metastasis and invasion by downregulating LYPD3 expression in HNSCC cells, the precise function of LYPD3 itself remains elusive. Moreover, notable disparity between the predicted molecular weight (~ 36 kD) and the observed band size (~ 75 kD) of LYPD3 protein in Western blot analyses prompted further investigation into its posttranslational modifications. Upon scrutinizing the protein structure of LYPD3, we identified four potential glycosylation sites in the protein [14], leading us to explore the significance of these modifications. To dissect the role of glycosylation, we introduced four mutations in the 118th, 163rd, 176th, and 183rd asparagine (N) residues, generate LYPD3 variants devoid of glycosylation sites, designated as LYPD3(4NA) (Fig. 3a). Western blotting results confirmed that abolishing glycosylation led to a substantial reduction in LYPD3 molecular weight to approximately 40 kD in HNSCC cells (Fig. 3b). Functional assays using Transwell assays unveiled that the migratory and invasive inhibitory effects of LYPD3 were nullified upon removal of glycosylation (Fig. 3c-e). Furthermore, immunofluorescence (IF) analysis revealed that glycosylated LYPD3 exhibited predominant localization within the cellular cytoplasm, while non-glycosylated LYPD3 displayed altered subcellular distribution patterns (Fig. 3f), potentially compromising its functional integrity. These findings collectively underscore the significance of LYPD3 glycosylation in orchestrating its subcellular localization and function, thereby influencing the metastatic progression of HNSCC.

### Upregulated miR-151-5p negatively correlates with LYPD3 expression and in HNSCC patients with metastasis

**Fig. 4:**
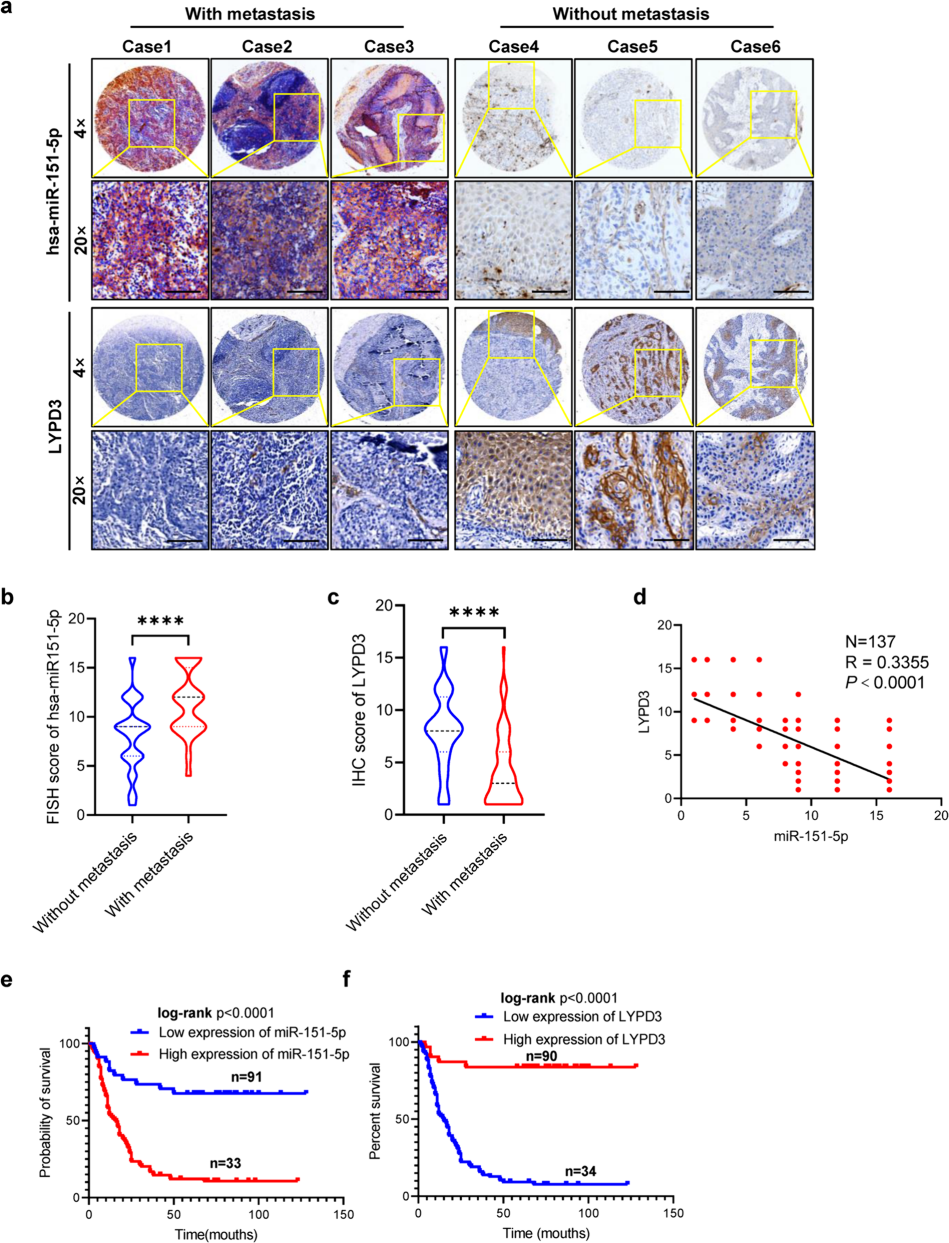
Upregulated miR-151-5p negatively correlates with LYPD3 expression and in HNSCC patients with metastasis. **a** Representative images of miR-151-5p in situ hybridization and immunohistochemical staining for LYPD3 on a tissue microarray consisting of 137 HNSCC tissues. **b**, **c** Quantitative statistical staining scores of miR-151-5p ISH and LYPD3 IHC in the HNSCC patient cohort according to whether the patients were confirmed to have cancer metastasis by Student’s t test. **d** The correlation between miR-151-5p expression and LYPD3 protein expression was investigated and analyzed in a tissue microarray (TMA) containing samples from 137 HNSCC patients by Pearson’s test. **e** Kaplan‒Meier plots illustrate that HNSCC patients with miR-151-5p levels above or equal to the median have shorter overall survival times than those with levels below the median. **f** Kaplan‒Meier plots illustrate that HNSCC patients with LYPD3 levels below the median experience shorter overall survival times than those with levels above the median. *P < 0.05, **P < 0.01, and ***P < 0.001 compared with the corresponding control

To evaluate the clinical relevance of the miR-151-5p/LYPD3 axis, we conducted in situ hybridization histochemistry (ISH) and immunohistochemistry (IHC) staining on a clinical cohort comprising 137 HNSCC specimens. Our findings revealed elevated miR-151-5p expression in HNSCC patients with cancer metastasis (Fig. 4a, b). Remarkably, patients exhibiting heightened miR-151-5p expression in this cohort displayed a poorer prognosis, with significantly shorter median survival times compared to those with lower miR-151-5p expression levels (Fig. 4e). Conversely, diminished expression of LYPD3 in HNSCC correlated with worse prognosis and higher frequency of metastasis (Fig. 4a, c). Intriguingly, patients with high miR-151-5p expression had a shorter median survival time than those with low miR-151-5p expression (Fig. 4f). Additionally, a conspicuous inverse correlation was observed between miR-151-5p expression and LYPD3 protein levels (Fig. 4d). Overall, these cohort results indicate that both miR-151-5p and LYPD3 as distinct biomarkers for prognostic evaluation in HNSCC patients.

### Modification of m6A mediates METTL3-induced pri-miR-151 maturation

**Fig. 5:**
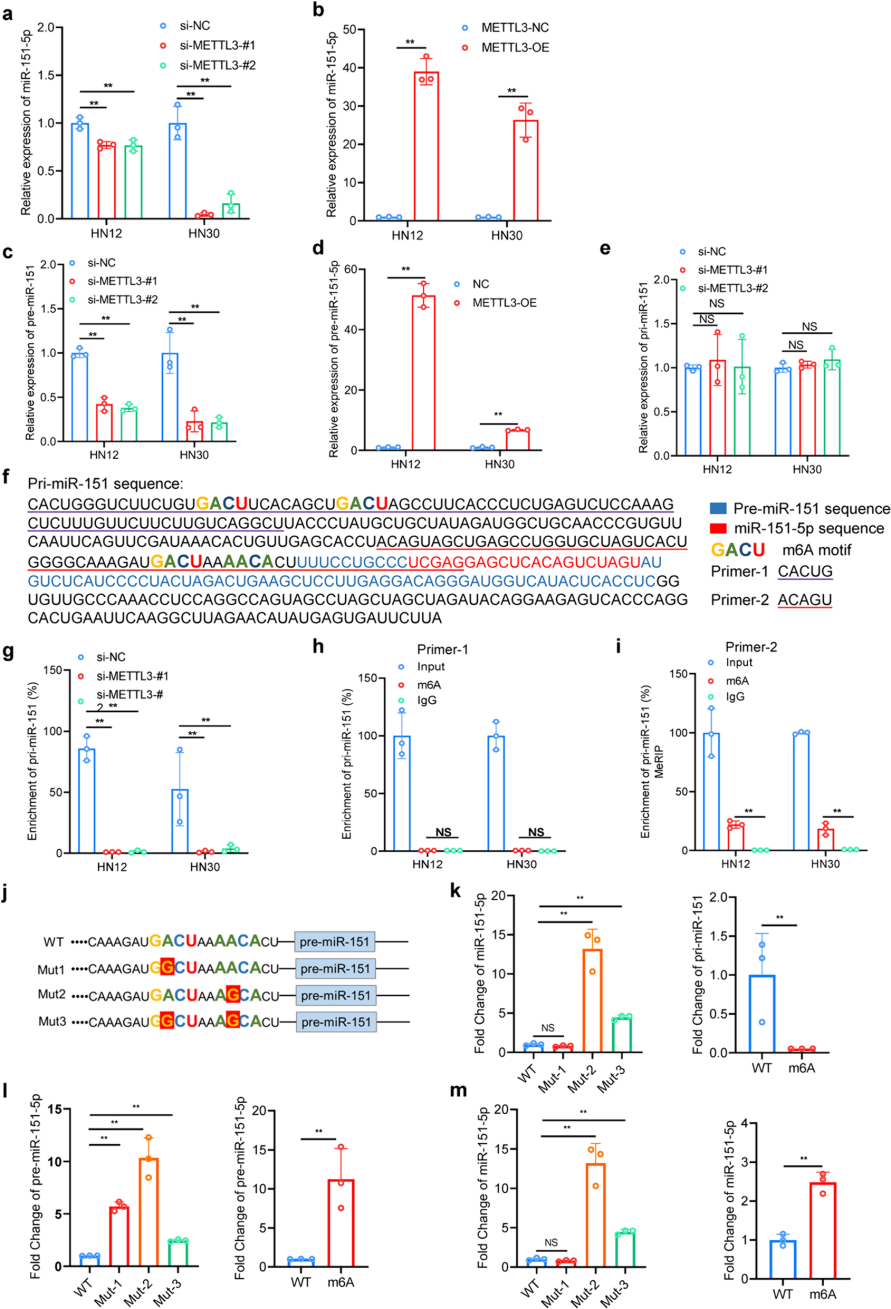
METTL3 mediates m6A modification of pri-miR-151 and promotes its maturation. **a** METTL3 knockdown by transfecting siRNA significantly decreased the expression of miR-151-5p in HNSCC cells. **b** Stable expression of METTL3 significantly increased the expression of miR-151-5p in HNSCC cells. **c** METTL3 knockdown by transfecting siRNA significantly decreased the expression of pre-miR-151 in HNSCC cells. **d** Stable expression of METTL3 significantly increased the expression of pre-miR-151 in HNSCC cells. **e** METTL3 knockdown by transfecting siRNA didn’t affect the expression of miR-151-5p in HNSCC cells. **f** The sequences of pri-miR-151, premiR-151, and miR-151-5p are indicated with distinct colors, with the m6A motif (GACU or AACA) positioned at the putative splicing site. Different primers used in the MeRIP assay are underlined in different colors. The sequence of Primer-1 and Primer-2 are underlined in purple and red relatively. **g** RIP assays with DGCR8 antibody demonstrate decreased enrichment of pri-miR-151 with METTL3 knockdown in HNSCC cells. **h**, **i** MeRIP assays with anti-N6-methyladenosine (m6A) antibody show the significant enrichment of pri-miR-151 detected with primer-2 in HNSCC cells. **j** Schematic representation of the mutation sites are indicated upstream of pre-miR-151. **k**-**m** Wild-type pri-miR-151 and mutant pri-miR-151 were synthesized using the T7-based MEGA shortscript kit. N6-methyl-ATP (m6A) was used as a replacement for ATP in the production of [m6A]pri-miR-151. In an in vitro reaction system, wild-type pri-miR-151, mutant pri-miR-151, or [m6A]pri-miR-151, along with total cellular lysates from 293 T cells transfected with plasmids expressing DROSHA and DGCR8, were used as starting materials. And m6A modification significantly enhanced the processing and maturation of pri-miR-151. pri-miR-1–1, which lacks a methylation site, served as a control. Quantification of pre-miR-151, miR-151-5p, and pri-miR-151 in the reaction mixture. Data represent fold change ± S.D. relative to pri-miR-151 WT. All statistical analyses in this figure are Student’s t test unless specified. *P < 0.05, **P < 0.01, and ***P < 0.001 compared with the corresponding control

The elevated levels of miR-151-5p across various HNSCC cells prompted our investigation into the underlying mechanisms. We hypothesized that m6A modification, a regulatory mark facilitating pri-microRNA (pri-miRNA) transcript processing, might be involved. Further investigation revealed that METTL3 significantly modulated the maturation of miR-151p. Specifically, overexpression of METTL3 increased both miR-151-5p (Fig. 5a, b) and pre-miR-151 (Fig. 5c, d) levels, while METTL3 knockdown led to a reduction in these levels. Unexpectedly, contrary to the e anticipated accumulation of pri-miRNAs upon METTL3 knockdown [15, 16], we observed no significant change in the level of pri-miR-151-5p (Fig. 5e). To delve deeper into these findings, we conducted RNA immunoprecipitation (RIP) in METTL3-silenced cells with an anti-DGCR8 antibody. The results unveiled a decrease in the interaction between pri-miR-151 and DGCR8 upon METTL3 knockdown (Fig. 5g), indicating a crucial role of METTL3 in facilitating miR-151-5p maturation. Subsequently, we explored the influence of m6A on miR-151-5p maturation. Four potential m6A motifs (GACU and AACU) were identified at the pri-miR-151 splicing site (Fig. 5f) based on m6A motif theory. Methylated RNA immunoprecipitation (MeRIP) assays targeting these motifs revealed significant enrichment of primer-2 in pri-miR-151 (Fig. 5h, i), suggesting its proximity to the pre-miR-151 m6A motif. To confirm the authentic m6A motif of pri-miR-151, we transcribed wild-type and mutant pri-miR-151 in vitro (Fig. 5j) and performed in vitro RNA processing assays using total cell lysates of 293 T cells overexpressing the microprocessor complex (DGCR8 and DROSHA). Results demonstrated that the mutation in GACU significantly impaired the efficiency of processing pri-miR-151 by the microprocessor complex compared to its wild-type and other mutated counterparts (Fig. 5k-m), hinting at a potential feedback mechanism regulating pri-miR-151 expression in living cells. Moreover, modifying the potential METTL3-catalyzing motif in pri-miR-151 with m6A significantly expedited pri-miR-151 processing to its mature forms(Fig. 5k-m), suggesting that heightened miR-151-5p levels in HNSCC may stem from augmented pri-miR-151 processing by METTL3-induced m6A modification.

### hnRNP U is a reader of m6A in pri-miR-151 maturation

**Fig. 6:**
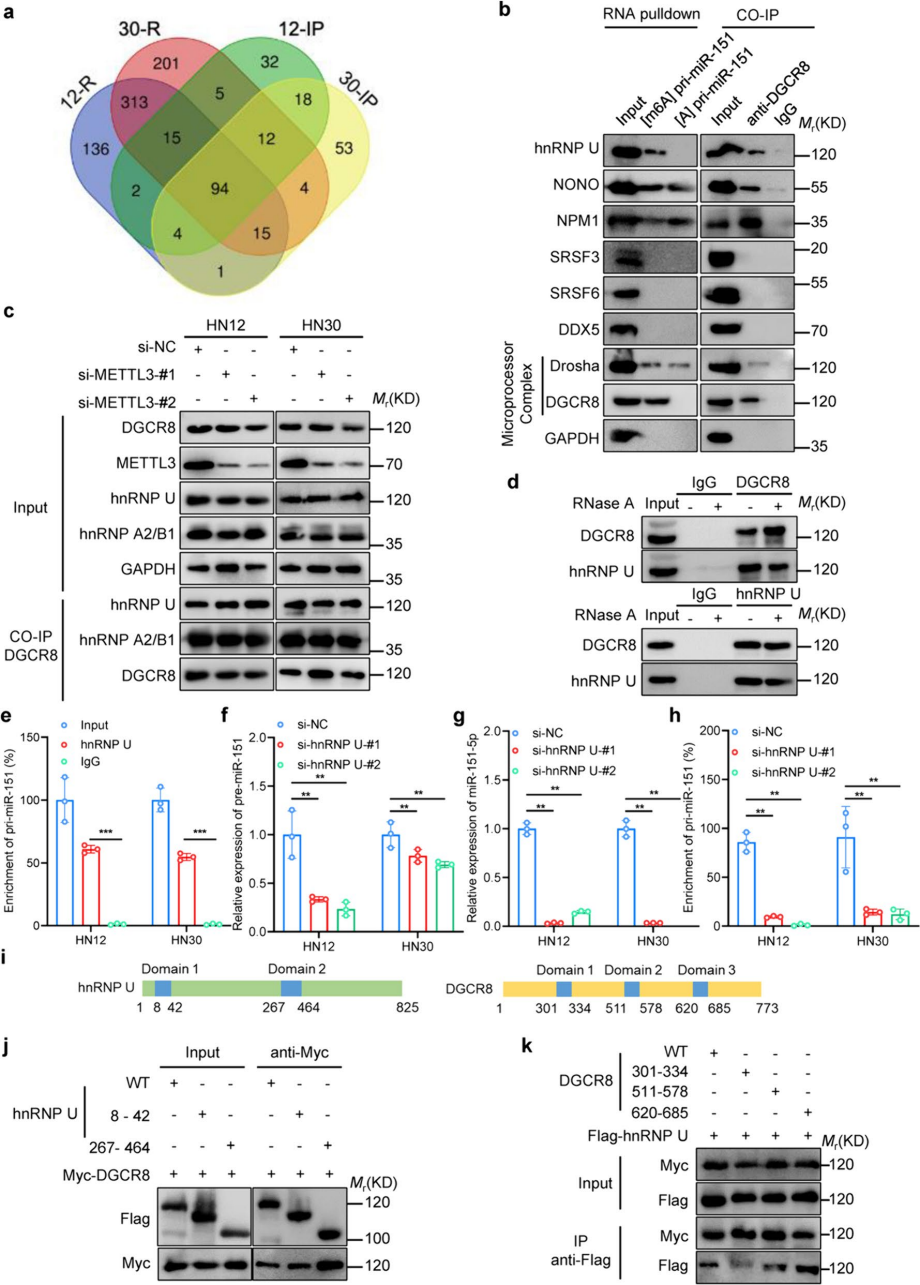
hnRNP U serves as a new m6A reader that promotes pri-miR-151 maturation by recruiting the microprocessor complex. **a** The schematic illustrates the approach used to identify potential readers pulled down by the [m6A]pri-miR-151 junction probe and coimmunoprecipitated by DGCR8 from HNSCC cells. 12-R and 30-R indicate RNA pulldown assays with [m6A]pri-miR-151 in HNSCC cells, respectively, and 12-IP and 30-IP indicate coimmunoprecipitation by DGCR8 in HNSCC cells, respectively. **b** Western blot analysis demonstrates the specific connection between hnRNP U and both [m6A]pri-miR-151 and DGCR8, as identified in the proteomic screening. **c** Coimmunoprecipitation assays with DGCR8 antibody reveal interactions between. DGCR8, hnRNP U and hnRNP A2/B1 in METTL3-knockdown HNSCC cells. **d** Reciprocal immunoprecipitation assays in 293 cells demonstrated the presence of RNase A didn’t affect the interaction between DGCR8 and hnRNP U. **e** RNA immunoprecipitation assays with hnRNP U antibody show enrichment of pri-miR-151 in HNSCC cells. **f** hnRNP U knockdown by transfecting siRNA significantly decreased the expression of pre-miR-151-5p in HNSCC cells. **g** hnRNP U knockdown by transfecting siRNA significantly decreased the expression of miR-151-5p in HNSCC cells. **h** RNA immunoprecipitation assays with DGCR8 antibody display a reduced enrichment of pri-miR-151 with hnRNP U knockdown in HNSCC cells. **i** The domain structural schematic of DGCR8 and hnRNP U proteins. **j**, **k** Immunoprecipitation assays were conducted on 293 T cells cotransfected with constructs expressing Myc-tagged DGCR8 (including wild-type and domain truncation mutants) and FLAG-tagged hnRNP U or FLAG-tagged hnRNP U (wild-type versus domain truncation mutants) and Myc-tagged DGCR8, respectively. All statistical analyses in this figure are Student’s t test unless specified. *P < 0.05, **P < 0.01, and ***P < 0.001 compared with the corresponding control

Recent studies have identified hnRNP A2/B1 as an m6A reader enhancing the processing of specific miRNAs, contingent on METLL3-mediated m6A modification [17]. However, for numerous other pri-miRNAs, including pri-miR-151, identity of m6A readers remains elusive. To address this gap, we conducted mass spectrometry analyses on proteins obtained through RNA pulldown via 50-bp pri-miR-151 or [m6A]pri-miR-151, followed by coimmunoprecipitation (co-IP) with an anti-DGCR8 antibody and subsequent integrative analysis (Fig. 6a). This approach identified 94 proteins, including 13 nuclear proteins potentially interacting with DGCR8 and [m6A]pri-miR-151 (Supplementary Tables 3 and 4). Of these 13 and proteins, only seven proteins were associated with RNA splicing. Further co-IP and RNA pull-down analyses revealed that among the seven nuclear proteins, only heterogeneous nuclear ribonucleoprotein U (hnRNP U) except for previously reported hnRNP A2/B1 bound both [m6A]pri-miR-151 and DGCR8 (Fig. 6b, c). Crucially, the interaction between hnRNP U and DGCR8 persisted even in the absence of METTL3 or in the presence of ribonuclease treatment (Fig. 6c, d). Additionally, we investigated the interaction between hnRNP U and pri-miR-151 through RIP assays, confirming a robust protein‒RNA association between hnRNP U and pri-miR-151 (Fig. 6e). Moreover, akin to METTL3 depletion, hnRNP U knockdown significantly reduced the interaction of endogenous DGCR8 with pri-miR-151 (Fig. 6f), suggesting that hnRNP U recruits DGCR8 to interact with pri-miR-151. Furthermore, hnRNP U knockdown significantly decreased the levels of premiR-151 and miR-151-5p in HNSCC cells, while the level of pri-miR-151-5p remained unchanged (Fig. 6g, h), indicated that hnRNP U promotes the maturation of miR-151-5p by recruiting the microprocessor complex (DGCR8 and DROSHA) in a m6A modification-dependent manner. Additionally, we explored whether hnRNP U interacts with DGCR8 via their functional domains (Fig. 6i). Through protein truncation mapping, we confirmed this interaction between hnRNP U and DGCR8, even after deleting the RNA-interaction domains (Fig. 6j, k). Collectively, these results strongly support the role of hnRNP U as both a m6A reader and a miRNA splicing-associated factor, shedding light on the intricate regulatory mechanisms underlying miRNA maturation in HNSCC.

## Discussion

**Fig. 7:**
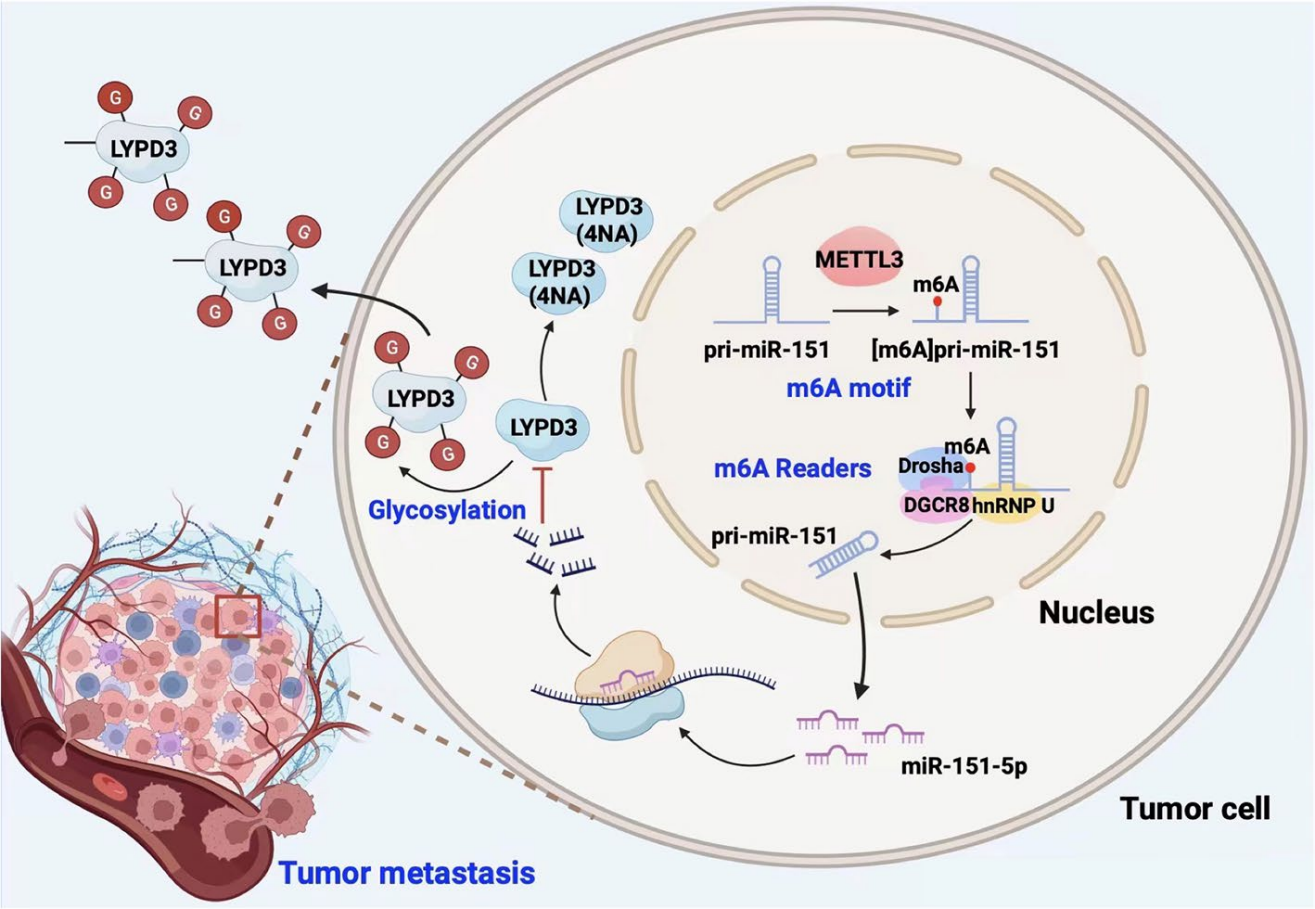
Mechanism schematic illustrating. A schematic illustrating the mechanism by which maturation of miR-151-5p via N6-methyladenosine, caused by METTL3 and mediated by hnRNP U, promotes migration and invasion of head and neck cancer by targeting LYPD3

HNSCC characterized by its aggressiveness and dismal prognosis, represents a formidable challenge in oncology [18]. Metastasis is a central contributing factor to the poor prognosis of HNSCC [10]. Despite the clinical urgency, the precise molecular mechanisms driving HNSCC metastasis remain elusive, and effective targeted therapies remain elusive [19, 20]. Hence, there is a pressing need to unravel the molecular underpinnings of HNSCC metastasis and identify potential therapeutic avenues. Here, our study presents compelling evidence implicating miR-151-5p overexpression in driving tumor metastasis and correlating with adverse prognosis in HNSCC. Mechanistically, we elucidate how m6A modification facilitates the maturation and accumulation of miR-151-5p, a process orchestrated by METTL3 and a newly identified m6A reader, hnRNP U. This culminates in the accumulation of miR-151-5p, which, in turn, propels the malignant progression of HNSCC by targeting LYPD3 (Fig. 7). This groundbreaking finding sheds light on the intricate workings of the METTL3/miR-151-5p/LYPD3 axis in augmenting the metastatic potential of HNSCC. These findings not only deepen our understanding of the molecular landscape of HNSCC metastasis but also offer promising therapeutic targets for intervention. By targeting key components of this regulatory axis, such as miR-151-5p, or LYPD3, novel therapeutic strategies may emerge to curb HNSCC metastasis and improve patient outcomes.

Posttranslational modification (PTM) of proteins is a fundamental process involving the chemical alteration of specific amino acid residues within proteins subsequent to their synthesis [21, 22]. Among these, glycosylation stands out as one of the most abundant intricate PTMs, significantly enriching the diversity of the proteome [23, 24]. Previous studies have demonstrated the presence of both N- and O-glycosylation on LYPD3 [14]. Our study provides compelling evidence highlighting the critical role of glycosylation in enhancing the function of LYPD3, ultimately leading to the suppression of metastasis and invasion in HNSCC cells. This effect is mediated through the modulation of LYPD3’s subcellular localization. Furthermore, the assessment of miR-151-5p and LYPD3 expression in the same cohort of HNSCC patients during a 5-year clinical follow-up has unveiled substantial prognostic significance in HNSCC. Previous research has extensively characterized LYPD3 as an oncogene, and its highly glycosylated cell surface protein has been associated with carcinogenic effects in various solid tumors [25–28]. Specifically, a study focused on HNSCC revealed that silencing LYPD3 led to reduced cell invasion and migration in CAL27 cells, effectively inhibiting the process of epithelial-mesenchymal transition. Furthermore, LYPD3 expression was markedly elevated in epithelial dysplasia and HNSCC tissues compared to normal oral mucosa samples [29]. Interestingly, our research has unveiled a contrasting role of LYPD3 in the malignancy of HNSCC cells and the clinical outlook for HNSCC patients. To address this experimental inconsistency, propose several explanations. Firstly, the divergence in cell models employed for investigating the function of LYPD3 could contribute to the observed dissimilarities. In addition, our comprehensive evaluation of LYPD3 expression and phenotypic traits across a spectrum of cell lines has indicated the plausible tumor-suppressive nature of LYPD3 in HNSCC cells. These findings open avenues for potential new approaches in the clinical diagnosis and treatment of metastatic HNSCC.

Furthermore, our study revealed that METTL3-mediated m6A modification on the primary transcript of miR-151-5p significantly enhances its splicing efficiency, thereby facilitating the rapid maturation and accumulation of miR-151-5p in HNSCC cells. Previous research has identified various nuclear proteins with potential roles as m6A readers, such as YTH family proteins [17, 30, 31], hnRNP A2/B1 protein [15], and, more recently, NKAP protein [16]. However, in our specific experimental context, we observed a distinct preference of hnRNP U for interacting with both [m6A]pri-miR-151 and DGCR8, thereby facilitating the maturation process of pri-miR-151. Initially identified as an mRNA splicer and a factor involved in packaging hnRNA into large ribonucleoprotein complexes, hnRNP U emerges in our study as a novel m6A reader specifically for pri-miR-151 [32]. This expands its functional repertoire from mRNA splicing and hnRNA packaging to facilitating pri-miRNA maturation [33, 34]. Further investigations revealed that the physical interaction between hnRNP U and DGCR8 occurs independently of m6A modification on pri-miRNA and even in the absence of RNA. Notably, hnRNP U lacks RNA splicing domains, including SAP and SPRY domains [34, 35], while DGCR8 lacks RNA splicing domains, including a WW domain and two DRBM1 domains, which interestingly, do not affect the interaction between hnRNP U and DGCR8. This indicates that the interaction between hnRNP U and DGCR8 is an intrinsic mechanism involved in the splicing of pri-miRNA into pre-miRNA. These findings highlight the intricate nature of m6A modification and its profound impact on pri-miRNA processing. Collectively, our results strongly suggest that the nuclear protein hnRNP U is a potential m6A reader in pri-miR-151. While the precise molecular mechanism underlying the association between [m6A]pri-miR-151, hnRNP U, hnRNP A2/B1, and DGCR8 warrants further elucidation, we hypothesize that hnRNP U recognizes the m6A mark and acts as a mediator, facilitating the interaction between [m6A]pri-miR-151 and DGCR8. This interaction, in turn, recruits the microprocessor complex to facilitate the maturation process.

It is imperative to acknowledge the limitations of our study. Despite employing multiple models and experiments to substantiate the significant role of the METTL3/miR-151-5p/LYPD3 axis in enhancing the metastatic potential of HNSCC, it is essential to recognize that this represents only one potential mechanism underlying tumor metastasis. Further investigation is warranted to fully elucidate the complexities of tumor metastasis. Although our study does not introduce innovation at the mechanistic level, it has yielded several noteworthy findings. For instance, we identified post-translational modifications of LYPD3 that may influence its function, as well as elucidated the expanded role of hnRNP U in RNA splicing, with implications for miRNA maturation and m6A processes. These findings present avenues for future research that hold significant promise.

Despite significant improvements in survival time and rates through local surgery for HNSCC patients [10], tumor recurrence and distant metastasis remain major causes of treatment failure after surgery [36]. Thus, understanding the mechanism of HNSCC metastasis holds crucial implications for clinical treatment. In this study, we discovered that METTL3 heightens m6A modification in pri-miR-151, expediting its maturation to miR-151-5p. Moreover, we observed that patients with tumor metastasis exhibited significantly higher miR-151-5p levels and worse prognoses than nonmetastatic patients. Conversely, LYPD3, the target gene of miR-151-5p, plays a contrasting role in HNSCC metastasis progression. Our findings strongly indicate that the METTL3/miR-151-5p/LYPD3 axis holds considerable promise as a prognostic biomarker and therapeutic target for HNSCC.

## Materials and methods

### Cell lines and cell culture

NOK cells (normal oral keratinocytes) and HOK cells (human oral keratinocytes) were nurtured in keratinocyte-SFM (Gibco). Cell lines (HEK293T, HN4, HN12, HN30, HN31, 1386Tu, 1386Ln, SCC25, Cal27) were cultivated in DMEM (Gibco, 11995073) supplemented with 10% FBS (Sigma, F0193) and 1% penicillin-streptavidin (Invitrogen, 15140148) and maintained in a humidified environment at 37 °C with 5% CO_2_. The provenances of all cell lines have been previously documented [37, 38].

### Plasmid construction, transfection and lentivirus production

All plasmids synthesized in this study were generated employing PrimeSTAR Max DNA Polymerase (Takara, R045A) and HiFi DNA Assembly Master Mix (NEB, E2621) via Gibson Assembly [39]. cDNAs encoding complete DGCR8, Drosha, hnRNP U, or truncated variants of DGCR8 and hnRNP U were subcloned and inserted into the pcDNA3.1 vector, incorporating Myc or Flag tags. Genes comprising METTL3, 3 × Flag-LYPD3, and mutated iterations of LYPD3, were subcloned and integrated into the pCDH-puro vector (SBI, CD510B-1). Transfection was carried out using Lipofectamine 2000 (Life Technologies). For lentivirus production, HEK293T cells were cotransfected with pCDH-based or miRNA-associated plasmids, alongside PsPAX2 and pMD2.g. After 48 h, the medium containing the lentiviral particles was filtered and employed for viral transduction.

### Quantitative real-time PCR analysis

Total RNA from the cell lines employed in this investigation was isolated using the RNeasy Mini kit (Qiagen, 74104). Genomic DNA was removed, and First-strand cDNA was synthesized using the PrimeScript RT Reagent Kit according to the instructions (Takara, RR047Q). Relative RNA expression was evaluated through triplicate qPCR analyses on a QuantStudio 3 Real-Time PCR System (Applied Biosystems) employing the SYBR Green approach. GAPDH and U6 small nuclear RNA were used as internal standards to quantify mRNA and miRNA levels, respectively. The relative RNA expression levels were computed using the comparative Ct methodology. The utilized primer sequences are listed in Supplementary Table 5.

### RNA interference

Small interfering RNA (siRNA) (listed in Supplementary Table 6) directed against the METTL3, hnRNP U, and LYPD3 genes, along with a nontargeting siRNA control, as well as miRNA mimics and inhibitors, were procured from RiboBio. Transfections involving siRNA or miRNA mimic and inhibitor were conducted using Lipofectamine RNAiMAX (Life Technologies, 13778100).

### Western blotting

For immunoblotting, all cellular samples were lysed using lysis buffer (comprising 2% SDS, 50 mM NaF, 10 mM sodium pyrophosphate, and 1.5 mM Na_3_VO_4_) supplemented with protease inhibitor cocktails (MCE, HY-K0010). Proteins in whole-cell lysates were separated using sodium dodecyl sulfate–polyacrylamide gel electrophoresis (SDS‒PAGE), transferred onto polyvinylidene difluoride (PVDF) membranes (Millipore, IPVH00010) by electroporation, and blocked with 5% nonfat milk. Then, the membranes were incubated with primary antibodies (listed in Supplementary Table 7) diluted in 5% bovine serum albumin (BSA) overnight at 4 ℃. This was followed by incubation with secondary antibodies diluted in 5% milk for 1 h at room temperature. Chemiluminescence detection was performed by applying ECL Western Blotting HRP Substrate (Millipore, WBKLS0500) to the membranes, which was captured using an imaging system.

### Immunoprecipitation and coimmunoprecipitation

Cells, either transfected with plasmids or not, were prepared using a gentle lysis buffer (containing 1 mM EDTA, 1% NP-40, 10 mM sodium pyrophosphate, 10 mM glycerophosphate, 50 mM HEPES at pH 7.5, 50 mM NaF, 150 mM NaCl, and 1.5 mM Na_3_VO_4_) supplemented with 1 mM PMSF and protease inhibitor cocktails (MCE, HY-K0010). Subsequently, they were incubated with antibodies (refer to Supplementary Table 7) overnight at 4 ℃. Protein A/G agarose beads (Beyotime, P2055) were introduced and incubated for more than 1 h to capture the protein-antibody complexes. Afterward, the beads underwent four wash cycles with gentle lysis buffer, and the proteins that were precipitated were solubilized in SDS‒PAGE sample buffer for subsequent analysis by immunoblotting.

### In vitro proliferation, invasion and migration assays

To conduct migration experiments, an 8 μm Transwell chamber (Corning, 3422) was placed in a 24-well plate. In addition, 200 μl of cell suspension was added to the upper chamber, where the concentration of HN30 cells was 7.5 × 10^5^ cells/ml and the concentration of HN12 cells was 2 × 10^4^ cells/ml. Complete DMEM was added to the lower chamber. After 24 h, the chamber was fixed with 4% paraformaldehyde (Sigma, 8187150100) and stained with 0.1% crystal violet (Sigma, C0775). Finally, slides were coated with neutral resin, and five fields of view were chosen under the microscope and quantified using ImageJ.

For invasion experiments, Matrigel (Corning, 356234) and serum-free medium were mixed at a 1:7 ratio, and 35 μl of this mixture was added to each chamber of the Transwell. The subsequent steps for the invasion experiment were the same as those described in the migration experiments. For cell proliferation assays, cells were seeded in 96-well plates at 2000 cells per well. After a specific incubation period, cell viability was evaluated via CCK-8 assays (Bioexplorer, B4007-016). Moreover, invasion study of 3D multicellular tumor spheroids was described previously [40–42], briefly, stable overexpression or knockdown of miR-151-5p on HN12 cell and HN30 cell were seed cells into 96-well round bottom Ultra-Low Attachment plate (Corning, No. 7007) at 5000 cells per well. Centrifuge the ULA plate (125 g,10 min) at room temperature. After incubated for 24 h, 100 µL of Matrigel (Corning, 356234) was slowly adding to wells for a final assay concentration of 50% in complete culture media. It was monitoring spheroid invasion by EVOS M5000 (Thermofisher) every 3–4 days.

### miRNA pull-down

Biotinylated miR-151-5p and control probes (obtained from RiboBio) were employed in our study. The experimental procedure was carried out utilizing the Pierce Magnetic RNA‒Protein Pull-Down Kit (Thermo, 20164). The pulled-down RNA was subsequently purified using the RNeasy MINI kit (Qiagen, 74104). The resulting product was then subjected to qPCR analysis and normalized with respect to the input.

### RNA immunoprecipitation and MeRIP assays

For RNA immunoprecipitation, cells were treated using a mild lysis buffer supplemented with RNase inhibitor (Thermo, AM2694). The resulting cell lysates were then subjected to an overnight incubation with antibodies (refer to Supplementary Table 7) at 4 ℃. For the MeRIP assay, RNA extracted from HNSCC cells was fragmented using RNA Fragmentation Reagents (Thermo, AM8740). Subsequently, appropriate amounts of fragmented RNA were subjected to an overnight incubation with antibodies (listed in Supplementary Table 7) at 4 ℃. The RNAs coprecipitated in both cases were quantified through qPCR analysis and normalized relative to the input.

### In vitro pri-miRNA processing assays

In our experiment, pri-miR-151 was synthesized using the T7-based MEGA shortscript kit (Life Technologies, AM1354). Notably, in the in vitro transcription reaction, N6-methyl-ATP (m6A) (Biorbit, orb65363) was used as a replacement for ATP and resulted in the production of [m6A]pri-miR-151. To assess pri-miR-151 processing, we incubated both pri-miR-151 and [m6A]pri-miR-151, along with pri-miR-1–1 (used as a control), with total cellular lysates obtained from 293 T cells that were cotransfected with plasmids carrying DROSHA and DGCR8. Subsequently, the total RNA extracted from the reaction products underwent analysis via qPCR. Furthermore, to investigate the impact of an A to G mutation at the m6A site of pri-miR-151 on its processing, mutant pri-miR-151 in vitro was synthesized by using the mutant template, and the in vitro verification method was the same as above. Primer sequences for this section are detailed in Supplementary Table 5.

### Animal experiments

To establish the pulmonary metastasis model, a total of twenty-four female BALB/c nude mice, aged between 4 and 5 weeks, were procured from Yaokang Biotechnology (China). The mice were randomly divided into three groups, each consisting of eight mice: HN30-CTRL, HN30-miR-151-5p-OE, and HN30-miR-151-5p-KD. Three distinct OSCC cell lines were prepared by achieving stable transduction with lentivirus. For each mouse, a mixture of 2 × 10^5^ cells suspended in 100 μL of normal saline was injected into the tail vein.

This experimental protocol was approved by the Ethics Committee of the West China Hospital of Stomatology, Sichuan University (WCHSIRB-D-2018–042). The experimental animals were maintained in a specific pathogen-free (SPF) breeding facility within the State Key Laboratory of Oral Diseases. Subsequent to lung extraction, assessments were conducted to evaluate pulmonary metastasis occurrence within each group. Hematoxylin–eosin (H&E) staining and immunohistochemistry (IHC) using anti-mitochondrial antibodies were carried out on the lung tissues to assess and characterize pulmonary metastasis.

### Tissue microarray

A total of 137 tissue samples were procured from individuals with head and neck squamous cell carcinoma (HNSCC) who underwent comprehensive surgical removal at the West China Hospital of Stomatology, Sichuan University (WCHSIRB-D-2018–042). These patients received regular follow-up assessments. During these follow-up visits, comprehensive clinical information and medical history were documented, encompassing factors such as age, sex, smoking habits, alcohol consumption, tumor differentiation, clinical TNM stage, and primary tumor location. The survival duration was meticulously recorded, beginning from the day of surgery and extending until either cancer-related death or the culmination of the 5-year follow-up period (Supplementary Tables 1 and 2). All tumor tissues and part of the adjacent normal tissues utilized for this research were acquired with the explicit informed consent of the patients. This study received ethical clearance and endorsement from the Ethics Committee of the West China Hospital of Stomatology, Sichuan University.

### Immunohistochemistry

Immunohistochemistry (IHC) experiments were conducted to detect LYPD3 using a primary antibody, following antigen retrieval with citrate buffer (0.01 M, pH 6.0). To evaluate the LYPD3 expression level within the HNSCC clinical cohort, a staining score was established. This score was determined by multiplying the staining intensity (1 for no staining, 2 for weak staining, 3 for moderate staining, and 4 for strong staining) by the staining area (1 for < 5% area, 2 for 5%-40% area, 3 for 41%-70% area, and 4 for 71%-100% area). This assessment was performed by two proficient pathologists who were unaware of the clinical and pathological details. To ascertain the prognostic significance within the HNSCC clinical cohort, the cumulative staining score was classified into two categories: low expression (staining score ranging from 1 to 8) and high expression (staining score ranging from 9 to 16).

### miRNA in situ hybridization

For miRNA in situ hybridization (ISH), a miRNA in situ hybridization kit from Boster Bio (China) was utilized. The tissue microarray was first subjected to routine deparaffinization and hydration. Subsequently, the tissue microarray was incubated with proteinase K for 30 min at 37 ℃ to facilitate miRNA accessibility. After that, the tissue microarray was washed in 2 × SSC solution and then stained with digoxigenin-labeled miR-151-5p probe overnight at 37 ℃. For signal detection, the tissue microarray was incubated with HRP-anti-digoxigenin secondary antibodies at 37 ℃ for 1 h. Finally, cover slides were treated with DAB (3,3’-diaminobenzidine) to visualize the hybridization signal, and the tissue sections were counterstained with hematoxylin for better visualization of the tissue structures.

### Immunofluorescence

Cells cultured on coverslips were first fixed using PBS containing 4% formaldehyde for a duration of 10 min. Subsequently, they underwent treatment with 0.1% Triton X-100 for 15 min. Following this, the cells were blocked using 3% goat serum and then incubated overnight at 4 ℃ with primary antibodies in PBST containing 3% BSA. Subsequently, the cells were exposed to secondary antibodies at room temperature for 1 h. Following these steps, coverslips were mounted using ProLong Gold Antifade Mountant with DAPI (Thermo Fisher, P36935). Images were captured using a confocal microscope.

### RNA sequencing and analysis

Following the treatment of cells transfected with miR-151-5p mimic or inhibitor, RNA samples were extracted using the RNeasy Mini kit (Qiagen, 74104). Subsequently, RNA-Seq and sequencing analysis were carried out by Novogene Company (China). As per the information provided by the company, genes exhibiting a false discovery rate (FDR) lower than 0.05 were considered to exhibit significant differences. The mRNA-seq data have been archived in the Gene Expression Omnibus repository under the accession number SUB13729846 and SUB14450220.

### Public data processing

Kaplan–Meier analysis using TCGA data was conducted utilizing the Kaplan‒Meier plotter tool (http://kmplot.com/analysis/). In silico analysis of miRNA target genes was carried out using four publicly accessible algorithms: miRCode, miRDB, miRTarBase, and TargetScan.

### Quantification and statistical analysis

Statistical analyses were carried out using GraphPad Prism 8.2 software (GraphPad Software, Inc., USA) or R. Each experiment was performed a minimum of three times, as indicated in the figures and corresponding figure legends. The results are presented as the mean ± standard error of the mean (SEM). For most datasets, statistical significance was determined using one-way ANOVA. When comparing two groups, Student’s t test was employed. The levels of significance were denoted as follows: *p < 0.05, **p < 0.01, ***p < 0.005, ****p < 0.001, and n.s. (not significant) when there was no significant difference observed.

### Supplementary Information

Supplementary Material 1.

## Data Availability

The raw RNA sequencing data have been archived in the Gene Expression Omnibus repository under the accession number SUB13729846 and SUB14450220. The comprehensive clinical information and medical history of 137 HNSCC patients such as age, sex, smoking habits, alcohol consumption, tumor differentiation, clinical TNM stage and primary tumor location were stated in Supplementary Tables 1 and 2. And any additional information required to reanalyze the data reported in this paper is available from the lead contact upon request.
