# Supplementary material for: m6A-dependent mature miR-151-5p accelerates the malignant process of HNSCC by targeting LYPD3
Source: Mol Biomed. 2024 Jul 16;5:27. doi: 10.1186/s43556-024-00189-9 (PMC11250566; doi:10.1186/s43556-024-00189-9)

m6A-Dependent Mature miR-151-5p Accelerates the Malignant Process of HNSCC by Targeting LYPD3

Huang *et.al*

Supplementary Table 1. Correlation of hsa-miR-151-5p in HNSCC with clinicopathological factors including patients and tumor characteristics.

Supplementary Table 2. Correlation of LYPD3 in HNSCC with clinicopathological factors including patients and tumor characteristics.

Supplementary Table 3. 94 proteins identified by RNA pulldown or immunoprecipitation assays followed by mass spectrometry analysis.

Supplementary Table 4. [m6A] pri-miR-151 and DGCR8 interacting nuclear proteins identified by RNA pulldown or immunoprecipitation assays followed by mass spectrometry analysis.

Supplementary Table 5. Primers used for quantitative real time-PCR and other assays in this study.

Supplementary Table 6. Sequences of siRNAs used in this study.

Supplementary Table 7. Antibodies used in this study.

Supplementary Fig. 1: related to Figure 1

Supplementary Fig. 2: related to Figure 2

Supplementary Fig. 3: related to Figure 2

Supplementary Fig. 4: related to Figure 5

Supplementary Fig. 5-14. Unprocessed gel blot.

Supplementary table 1. Correlation of hsa-miR-151-5p in HNSCC with clinicopathological factors including patients and tumor characteristics.

**Characteristics**	**number of cases**	**miR-151-5p (Low)**	**miR-151-5p (High)**	***p* value**
Age at surgery				
≤60 years	62	13	49	
60 years	75	24	51	0.1778
Gender				
Male	99	25	74	
Female	38	12	26	0.5205
Smoking				
No	65	18	47	
Yes	72	19	53	> 0.9999
Alcohol consumption				
No	59	15	44	
Yes	78	22	56	0.8462
Differentiation				
High	82	25	57	
Moderate	40	9	31	
Poor	15	3	12	
High vs. moderate + poor				0.3276
Tumor stage				
T1	24	6	18	
T2	59	22	37	
T3	18	3	15	
T4	36	6	30	
T1-2 vs T3-4				0.0314
Clinical TNM stage				
I	14	5	9	
II	31	16	15	
III	35	9	26	
IV	57	7	50	
I-III vs IV				0.0010
Lymph node metastasis				
No	65	29	36	
Uniateral	65	8	57	
Bilateral	7	0	7	
No vs. unilateral + bilateral				<0.00011

P values were derived using the Spearman rank correlation coefficient test; all statistical tests are two-sided.

The expression levels of hsa-miR-151-5p were significantly correlated with tumor stage ($P = 0.0314$), clinical TNM stage ($P = 0.0010$) and lymph node metastasis ($P < 0.0001$) of HNSCC according to our clinical cohort (137 cases of HNSCC).

Supplementary table 2. Correlation of LYPD3 in HNSCC with clinicopathological factors including patients and tumor characteristics.

**Characteristics**	**number of cases**	**LYPD3 (Low)**	**LYPD3 (High)**	***p* value**
Age at surgery				
≤60 years	62	52	10	
60 years	75	52	23	0.0700
Gender				
Male	99	75	24	
Female	38	29	9	>0.999
Smoking				
No	65	51	14	
Yes	72	53	19	0.5528
Alcohol consumption				
No	58	46	12	
Yes	79	58	21	0.5447
Differentiation				
High	82	56	26	
Moderate	40	36	4	
Poor	15	12	3	
High vs. moderate + poor				0.0140
Tumor stage				
T1	24	20	4	
T2	59	42	17	
T3	18	13	5	
T4	36	29	7	
T1-2 vs T3-4				0.8383
Clinical TNM stage				
I	14	10	4	
II	31	19	12	
III	35	26	9	
IV	57	49	8	
I-III vs IV				0.0255
Lymph node metastasis				
No	65	40	25	
Uniateral	65	57	8	
Bilateral	7	7	0	
No vs. unilateral + bilateral				0.0003

P values were derived using the Spearman rank correlation coefficient test; all statistical tests are two-sided.

The expression levels of LYPD3 were significantly correlated with differentiation (*P* = 0.0140), clinical TNM stage (*P* = 0.0255) and lymph node metastasis (*P* = 0.0003) of HNSCC according to our clinical cohort (137 cases of HNSCC).

Supplementary table 3. 94 proteins identified by RNA pulldown or immunoprecipitation assays followed by mass spectrometry analysis.

**Protein name**							
ACTB	FILA2	K1C14	RL10	RL22	RL36	RS14	RS4X
ALBU	H13	K1C9	RL10A	RL23	RL36L	RS15A	RS6
ANXA2	H15	K22E	RL11	RL23A	RL4	RS16	RS7
ATPA	H2B2F	K2C1	RL12	RL24	RL6	RS18	RS8
BIP	H33	K2C5	RL13	RL26	RL7	RS19	RS9
DCD	H4	K2C8	RL13A	RL27	RL7A	RS2	S10A9
DDX5	HNRPU	LRC59	RL14	RL27A	RL8	RS23	SRSF3
DESP	HORN	NONO	RL15	RL28	RL9	RS24	SRSF6
DSG1	HS71B	NPM	RL17	RL3	RLA0	RS25	SYDC
EF1A1	HSP7C	NUCL	RL18	RL30	ROA2	RS26	XRCC5
EF1G	HSPB1	PAIRB	RL18A	RL31	RS11	RS3	
ENOA	K1C10	PIP	RL19	RL34	RS13	RS3A	

Supplementary table 4. [m6A] pri-miR-151 and DGCR8 interacting nuclear proteins identified by RNA pulldown or immunoprecipitation assays followed by mass spectrometry analysis.

**Protein Name**	**Gene Name**	**NCBI-accession**
Heterogeneous Nuclear Ribonucleoprotein U (hnRNP U)	hnRNP U	Q00839
Heterogeneous Nuclear Ribonucleoprotein A2/B1(hnRNP A2/B1)	hnRNP A2/B1 or ROA2	P22626
Non-POU Domain Containing Octamer Binding (NONO)	NONO	Q15233
Nucleophosmin 1(NPM1)	NPM1	P06748
Serine And Arginine Rich Splicing Factor 3(SRSF3)	SRSF3	P84103
Serine And Arginine Rich Splicing Factor 6(SRSF6)	SRSF6	Q13247
DEAD-Box Helicase 5 （DDX5）	DDX5	P17844
Annexin A2	ANXA2	P07355
Alpha-enolase	ENO1	P06733
Leucine-rich repeat-containing protein 59	LRC59	Q96AG4
Plasminogen activator inhibitor 1 RNA-binding protein	PAIRB	Q8NC51
Prolactin-inducible protein	PIP	P12273
X-ray repair cross-complementing protein 5	XRCC5	P13010

Supplementary Table 5. Primers used for quantitative real time-PCR and other assays in this study.

Gene Symbol	Forward primer (5'→3')	Reverse primer (5'→3')
LYPD3	GATGCTCCCCGAACAAGATGA	CAGCGAGAATTGTCCGTGGAT
URGCP	GACCTTGCTGCCGACATTTAT	GCAGGAAACTGTCTGAGGAGAG
GAPDH	GGAGCGAGATCCCTCCAAAAT	GGCTGTTGTCATACTTCTCATGG
hsa-pri-miR-1	TATGTATCTCAGGCCGGGACC	GGACACGACCGTCCACCA
hsa-pri-miR-151-#1	CACTGGGTCTTCTGTGACTT	AGCCTGACAAGAAGAACAAA
hsa-pri-miR-151-#2	ACAGTAGCTGAGCCTGGTGC	CTCGAGGGCAGGAAAAGTGT
Reverse transcription primers		
hsa-miR-151-5p	GTCGTATCCAGTGCAGGGTCCGAGGTATTCGCACTGGATACGACACTAGA	
hsa-miR-1-1-3p	GTCGTATCCAGTGCGTGTCGTGGAGTCGGCAATTGCACTGGATACGACATACATAC	
Stem-loop RT-PCR primers		
hsa-miR-151-5p	CGCGTCGAGGAGCTCACAG	ATCCAGTGCAGGGTCCGAGG
hsa-miR-1-1-3p	ACGTGGAATGTAAAGAAGTATGTATGTC	ATCCAGTGCAGGGTCCGAGG
hsa-pre-miR-151	TTTCCTGCCCTCGAGGAGCTCACAG	GAGGTGAGTATGACCATCCCTGTCCTCAAG
hsa-pre-miR-1-1	GAAACATACTTCTTTATATGCCCAT	TTGCCCTATGTATGAAGAAATGTAAGGTAT
U6	CTCGCTTCGGCAGCACA	AACGCTTCACGAATTTGCGT

Supplementary Table 6. Sequences of siRNAs used in this study.

Sequences of siRNA	5'→3'
si-Control	UUCUCCGAACGUGUCACGUTT
METTL3-siRNA-#1	GGAGAUCCUAGAGCUAUUATT
METTL3-siRNA-#2	GCACAUCCUACUCUUGUAATT
LYPD3-siRNA-#1	AGGGCAGUAUCCUGCAAAATT
LYPD3-siRNA-#2	CUACAACGCCAGCGAUCAUTT
hnRNP U-siRNA-#1	GGAUUGUGAGGUUGUAAUGTT
hnRNP U-siRNA-#2	CAUUACAACCUCACAAUCCTT
miR-151-5p mimic	UCGAGGAGCUCACAGUCUAGU
miR-151-3p inhibitor	ACUAGACUGUGAGCUCCUCGA

Supplementary Table 7. Antibodies used in this study.

Antibodies	Source	Identifier
GAPDH	Cell Signaling Technology	5174
METTL3	Abcam	ab195352
Tubulin	Cell Signaling Technology	2125
N6-methyladenosine (m6A)	Abcam	ab284130
Drosha	Abcam	ab303544
DGCR8	Abcam	ab191875
AGO2	Abcam	ab186733
Mitochondria	Abcam	ab92824
hnRNP U	Abcam	ab180952
hnRNP A2B1	Abcam	Ab259894
DDX5	Novus	NB200-351
SRSF6	Abcam	140623
SRSF3	Abcam	198291
NPM1	Novus	AF5205
NONO	Novus	NB100-1556
LYPD3	Novus	AF5567
URGCP	Proteintech	11998-1-AP
E-cardherin	Cell Signaling Technology	14472S
Vimentin	Cell Signaling Technology	5741S
Myc-tag	Cell Signaling Technology	2278
Flag-tag	Sigma	F1804
Goat anti-mouse IgG antibody, Alexa Fluor 488	Thermo Fisher Scientific	Cat#A11001
Mouse IgG Isotype Control	Zenbio	A00001
Rabbit IgG Isotype Control	Zenbio	A00002

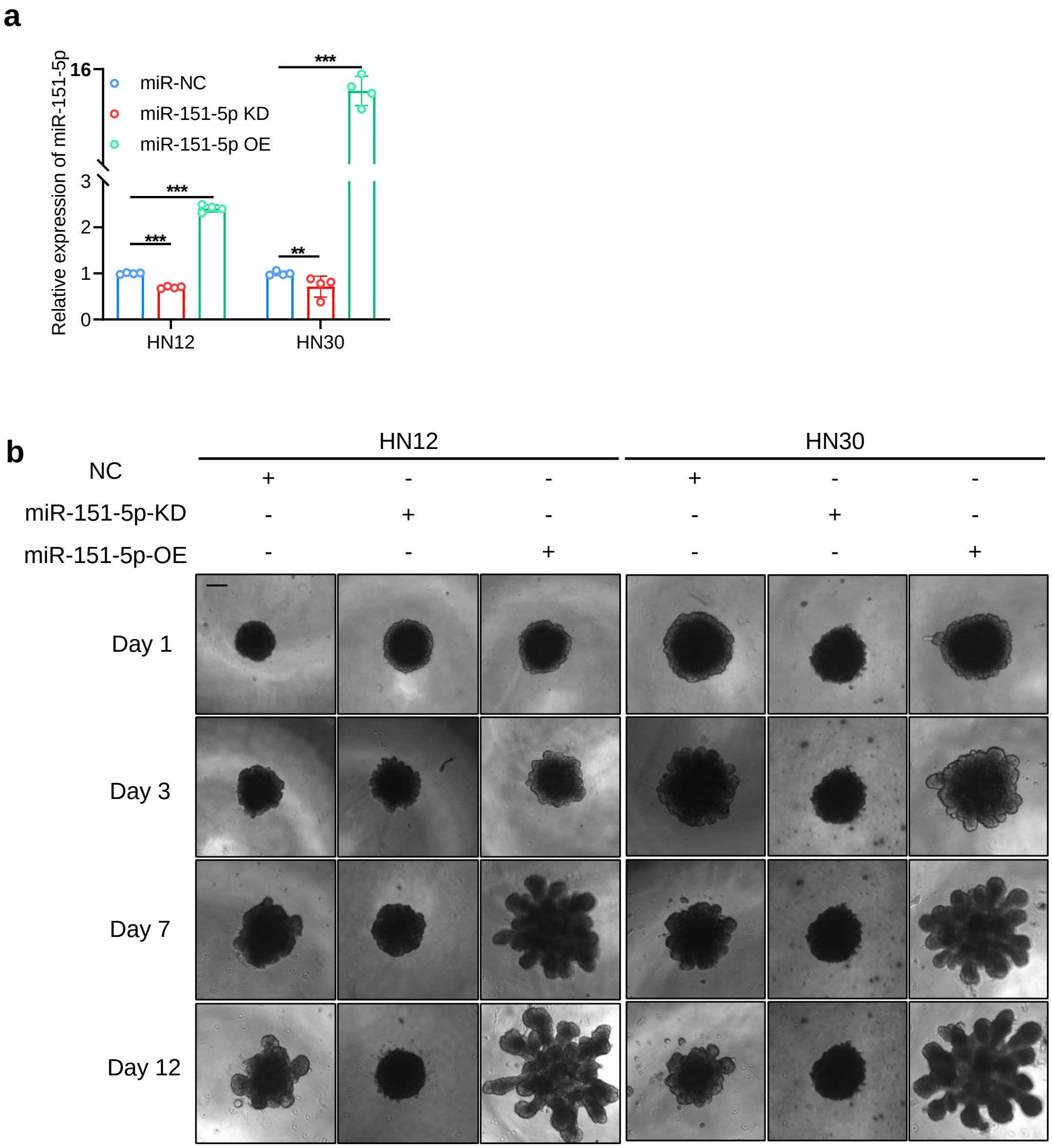

Figure S1: related to Figure 1: miR-151-5p is overexpressed in HNSCC cell lines and related to invasion and migration.

(a) Expression levels of miR-151-5p in HNSCC cells with knockdown or of overexpression miR-151-5p by lentivirus.

(b) The impact of stable overexpression or knockdown of miR-151-5p on the cell invasion ability of HNSCC cells by 3D multicellular tumor spheroids assay, with representative images.

Supplementary Fig.2

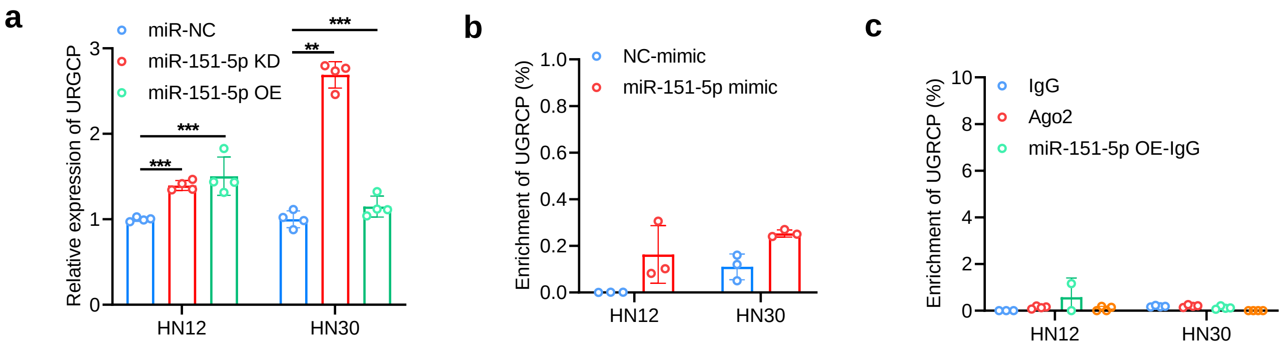

Figure S2: related to Figure 2: miR-151-5p inhibited the LYPD3 expression by binding to the LYPD3 mRNA 3'-UTR, resulting in the promotion of the invasion and migration of HNSCC cells.

(a) qRT-PCR reveled expression levels of URGCP mRNA in HNSCC cells with knocking down and overexpression of miR-151-5p

(b) miRNA pull-down assays with biotin labeling miR-151-5p mimic showed little enrichment levels of URGCP mRNA in HNSCC cells

(c) RNA immunoprecipitation assays with Ago2 showed little enrichment of URGCP mRNA in HNSCC cells overexpressing miR-151-5p.

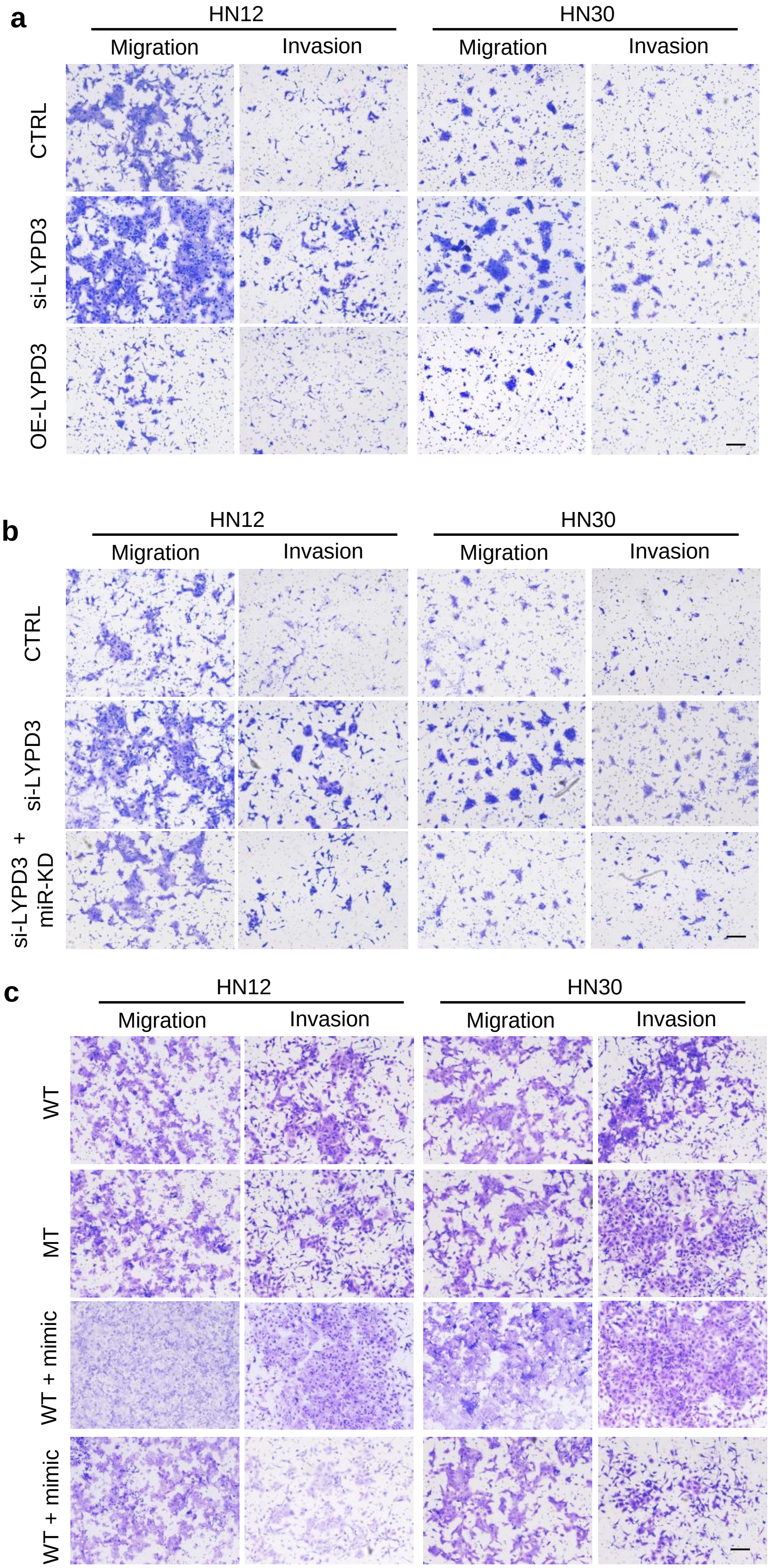

Figure S3: related to Figure 2 : miR-151-5p inhibited the LYPD3 expression by binding to the LYPD3 mRNA 3'-UTR, resulting in the promotion of the invasion and migration of HNSCC cells.

(a-c) Effects of treatment as mentioned in the figures on migration and invasion of HNSCC cells *in vitro,* which are representative pictures of transwell assays showing different abilities of cell migration and invasion. Scale bars, 200 μm.

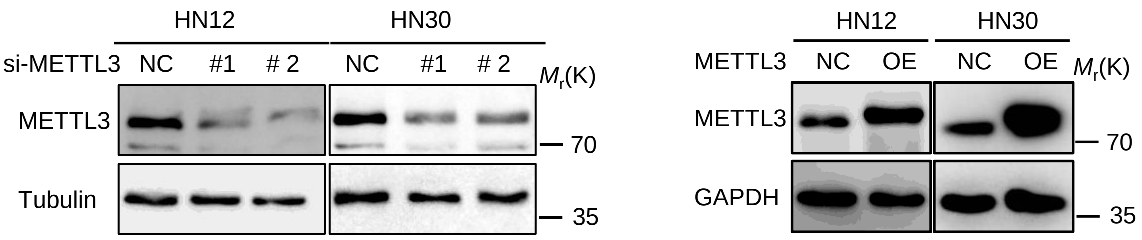

Figure S4: related to Figure 5: METTL3 mediates m6A modification of pri-miR-151 and promotes its maturation.

Western blot analysis of expression of METTLE with si-METTL3 transfection and overexpression by lentivirus.

Supplementary Fig. 5. Unprocessed gel blots.
Of note, for some immunoblotting assays membranes were cut into several pieces to incubate with different antibodies, and therefore the raw images of these membranes are of small size.

Figure 2B

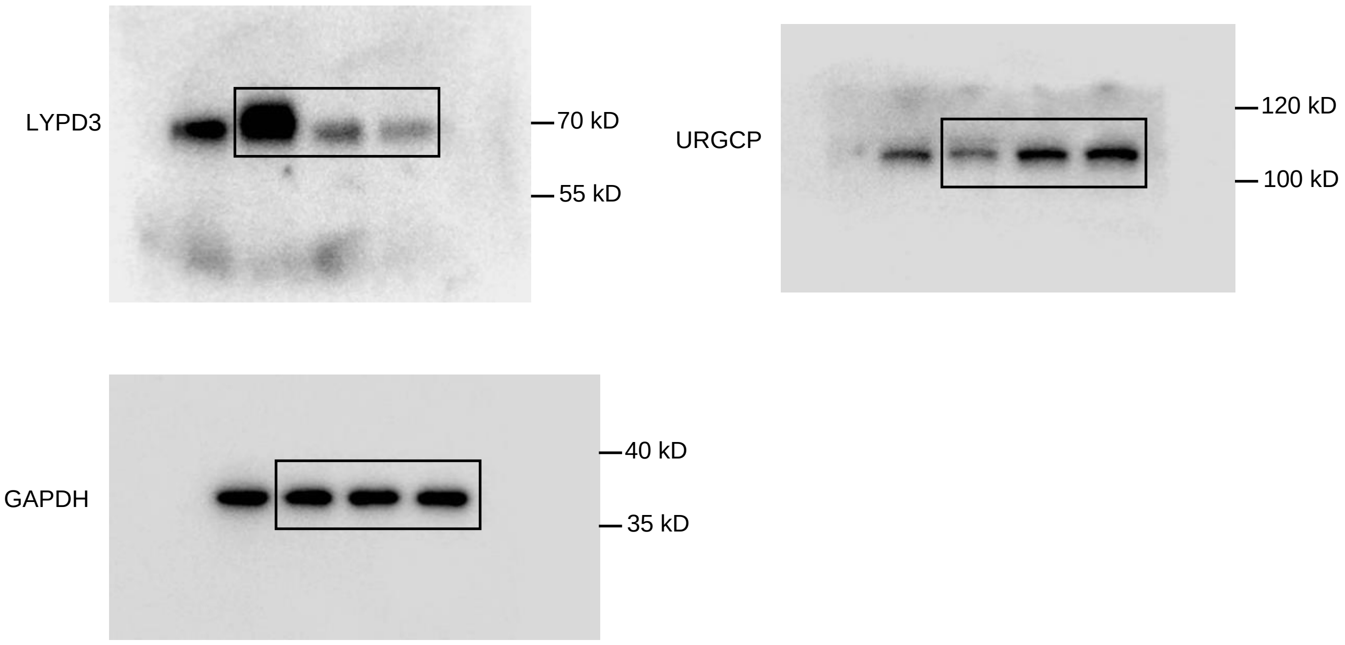

Supplementary Fig. 6. Unprocessed gel blots. (Continued)

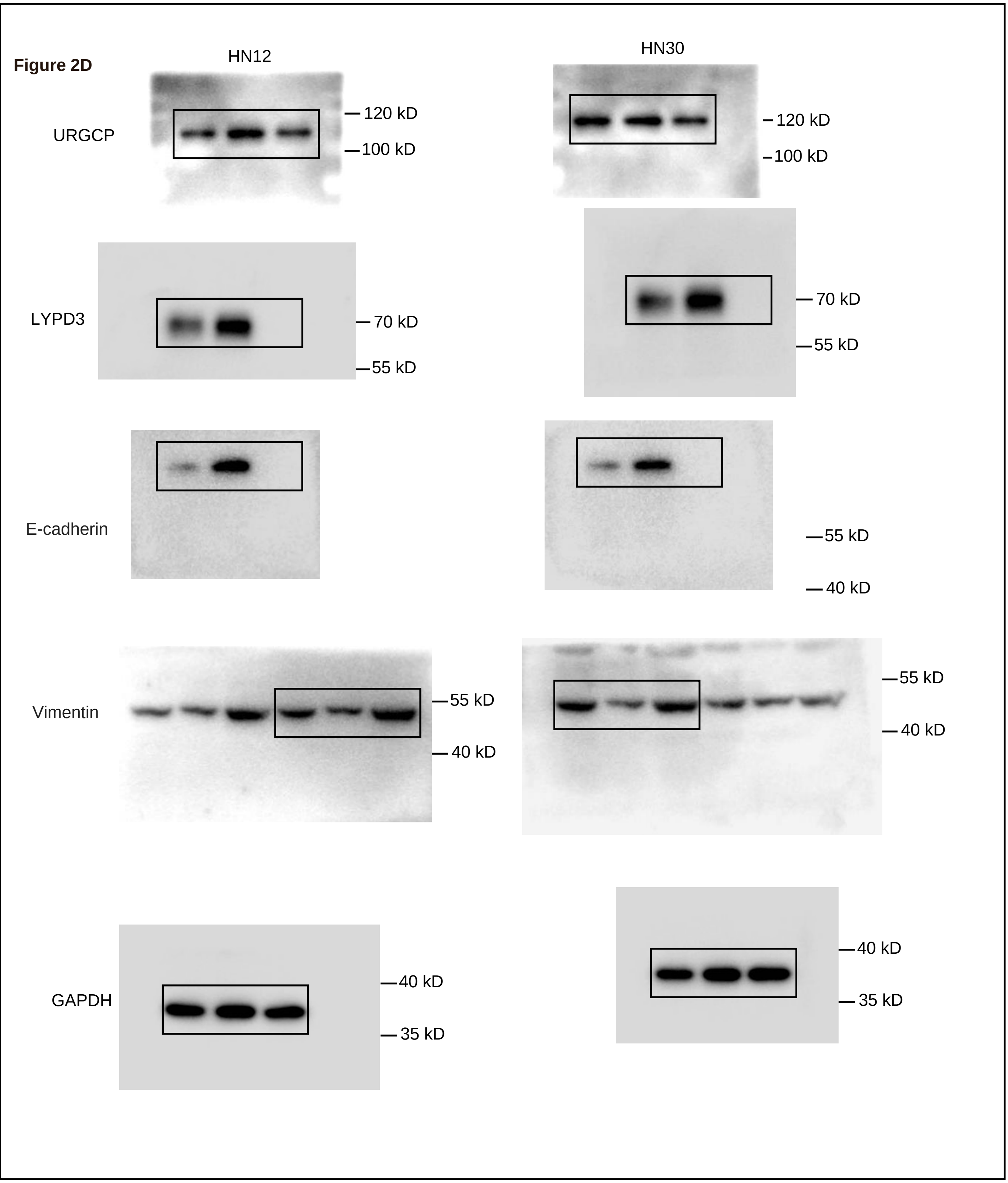

Supplementary Fig. 7. Unprocessed gel blots. (Continued)

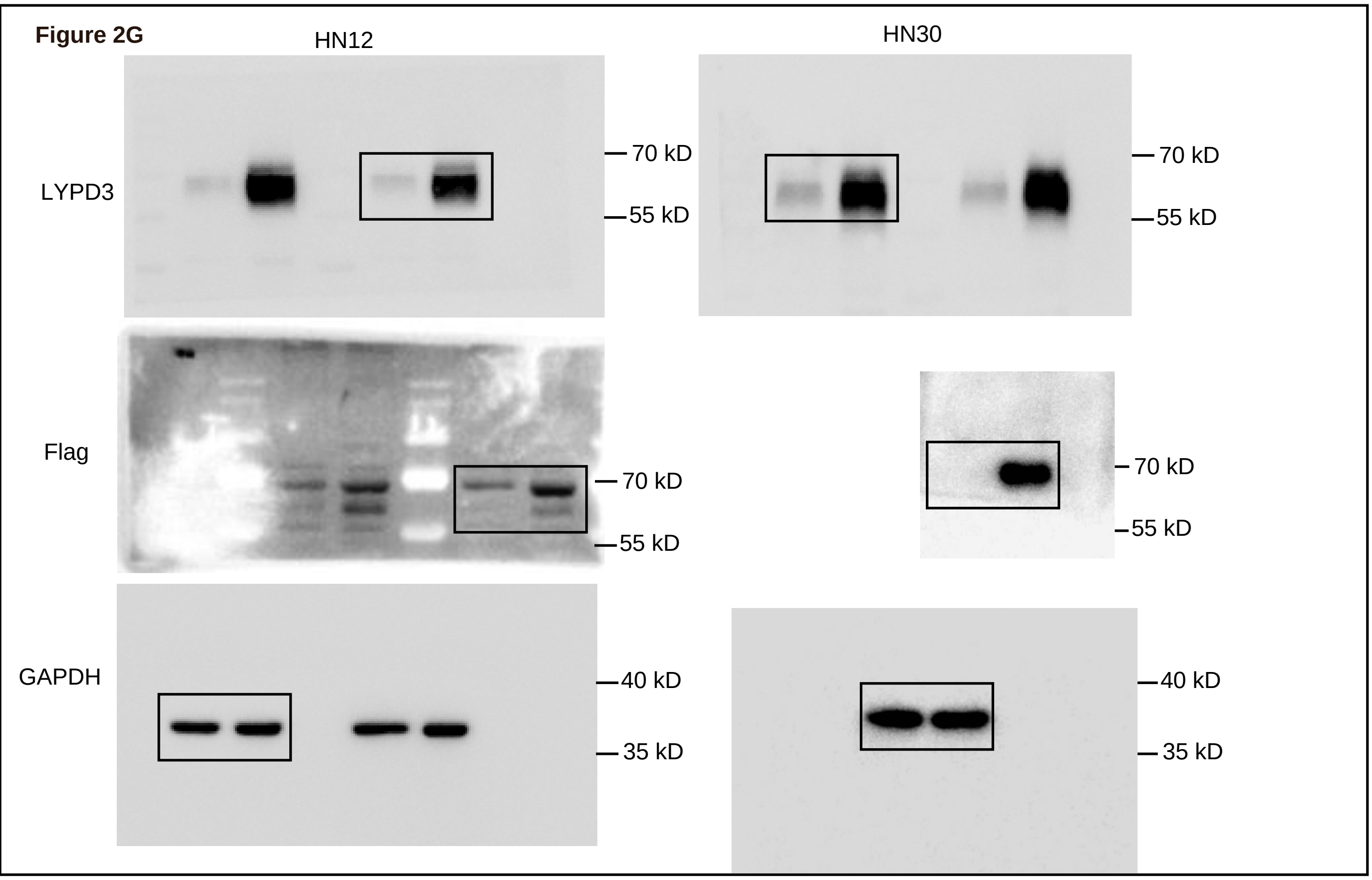

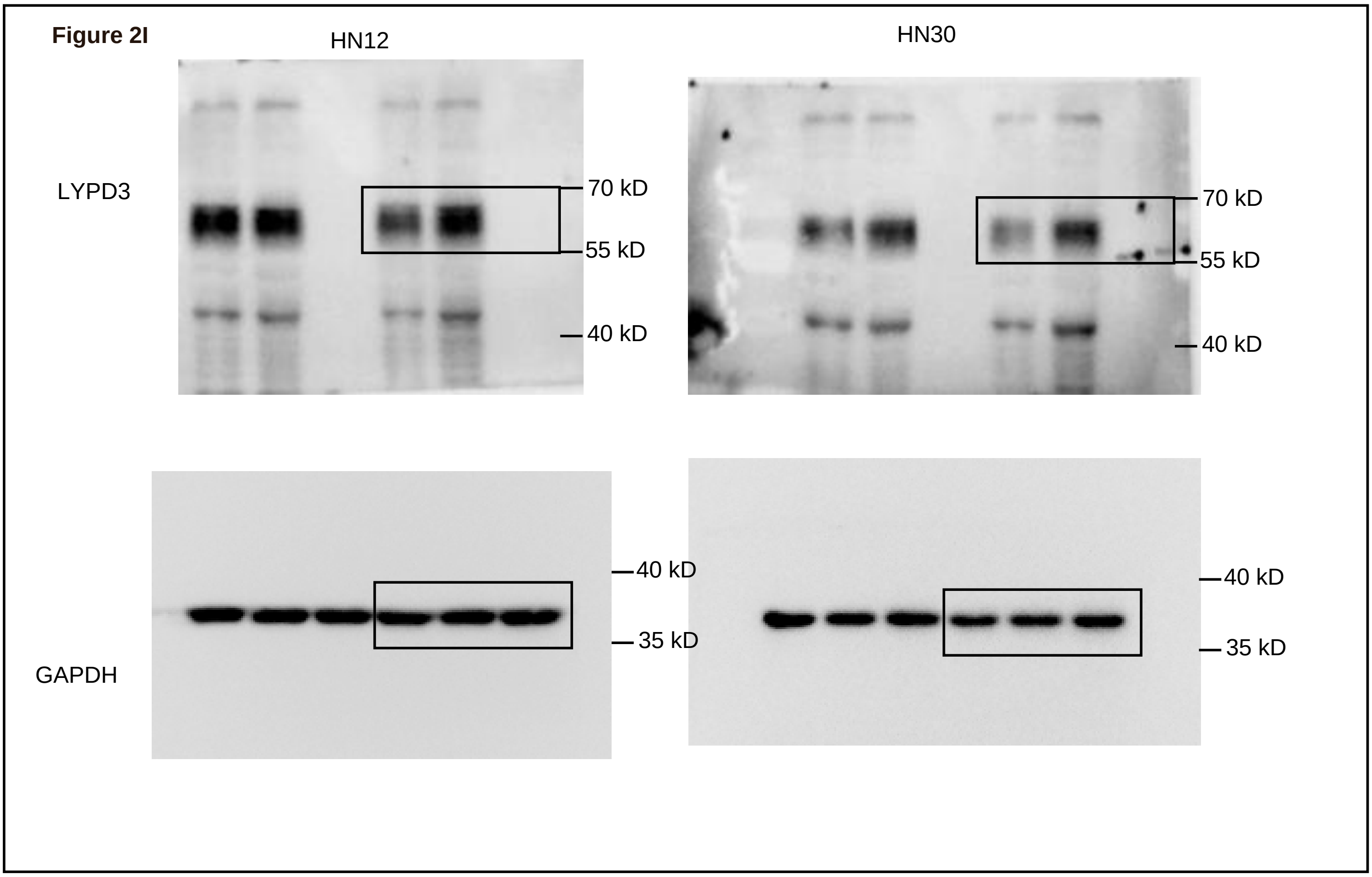

Supplementary Fig. 8. Unprocessed gel blots. (Continued)

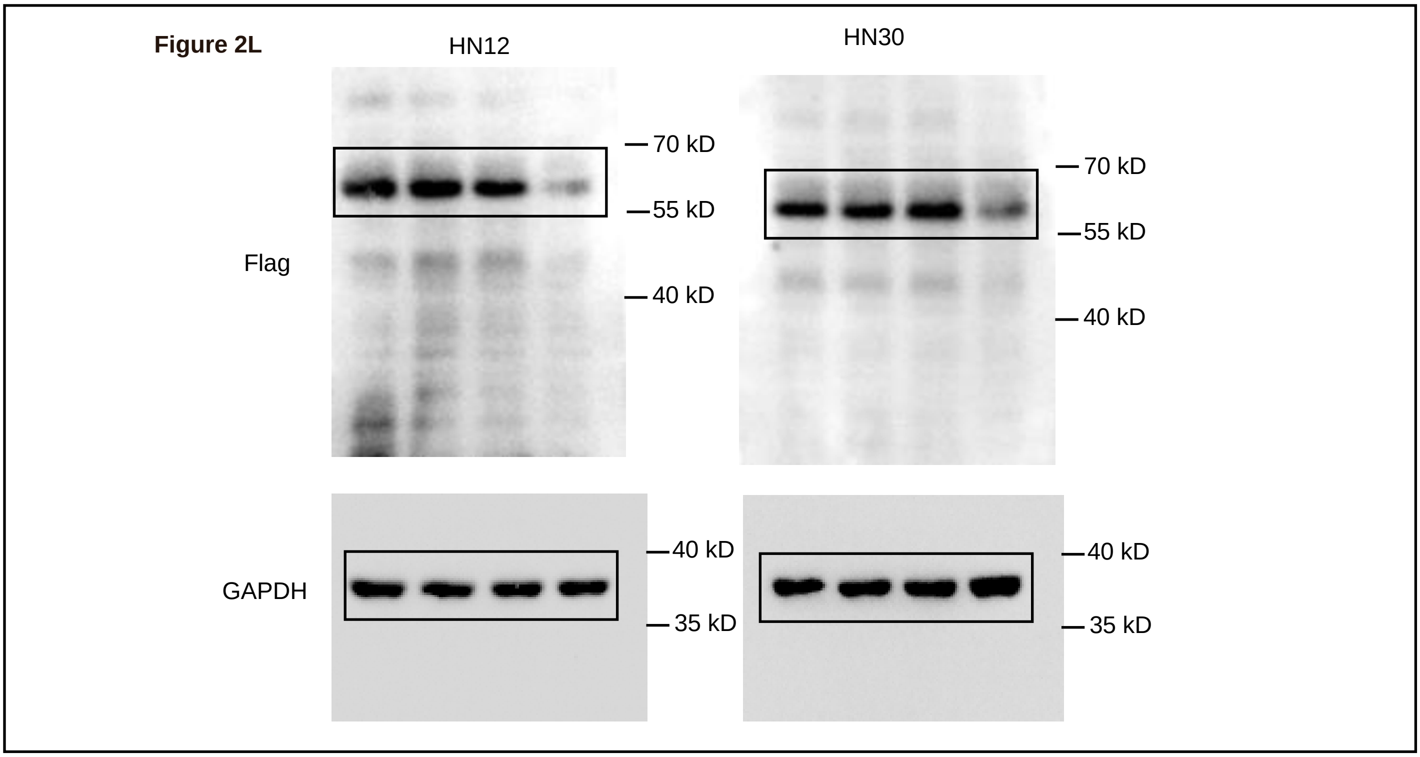

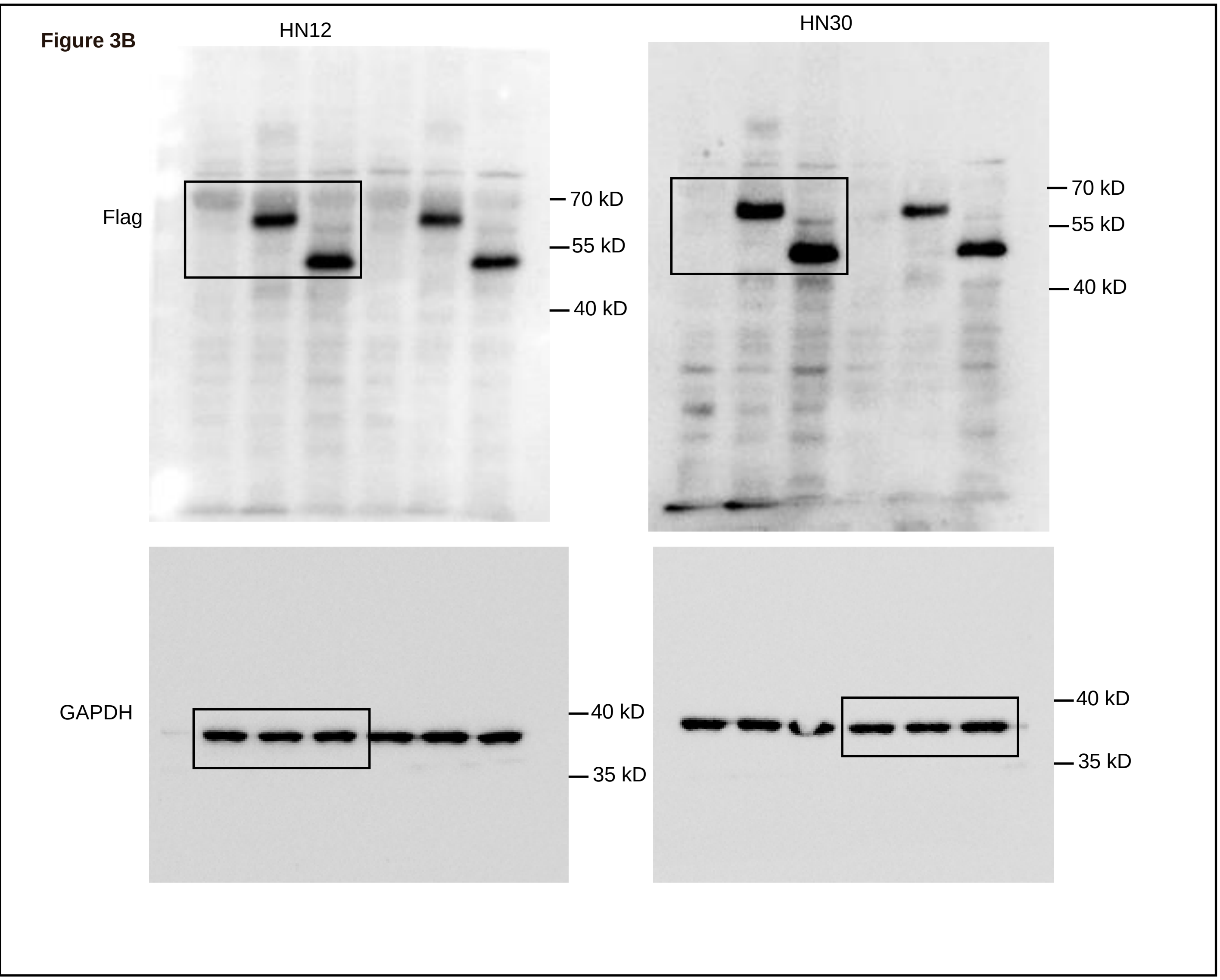

Supplementary Fig. 9. Unprocessed gel blots. (Continued)

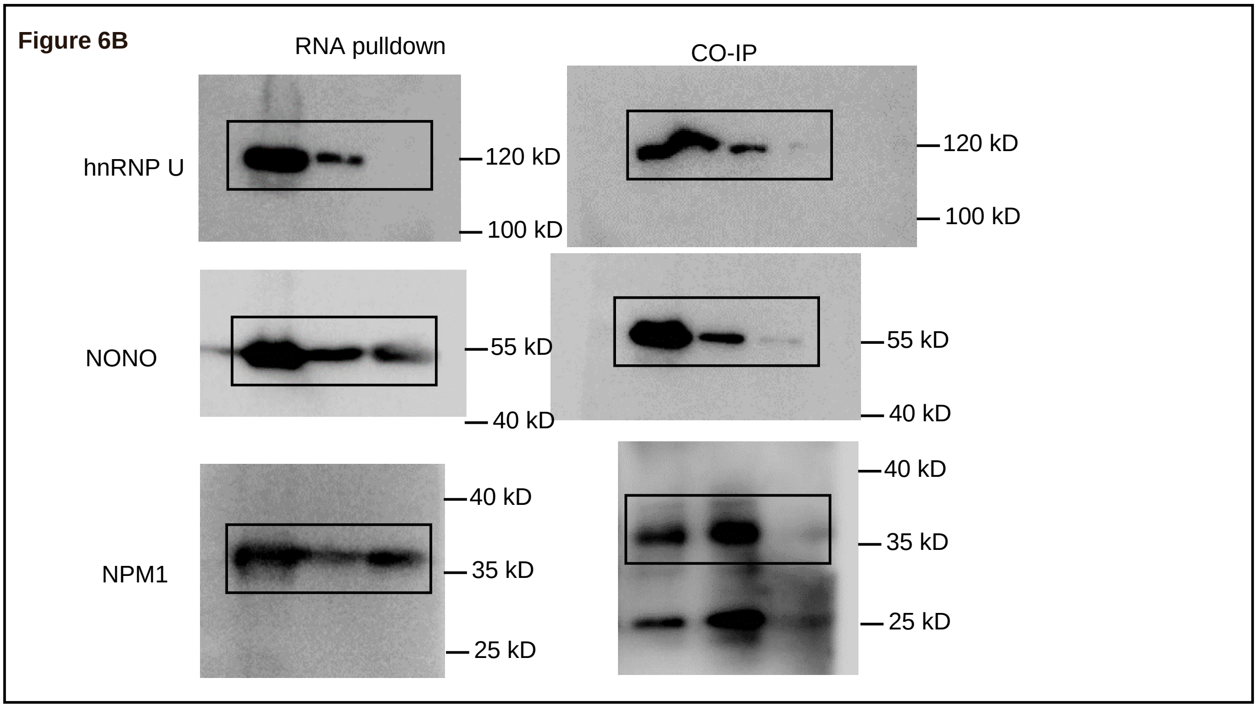

Supplementary Fig. 10. Unprocessed gel blots. (Continued)

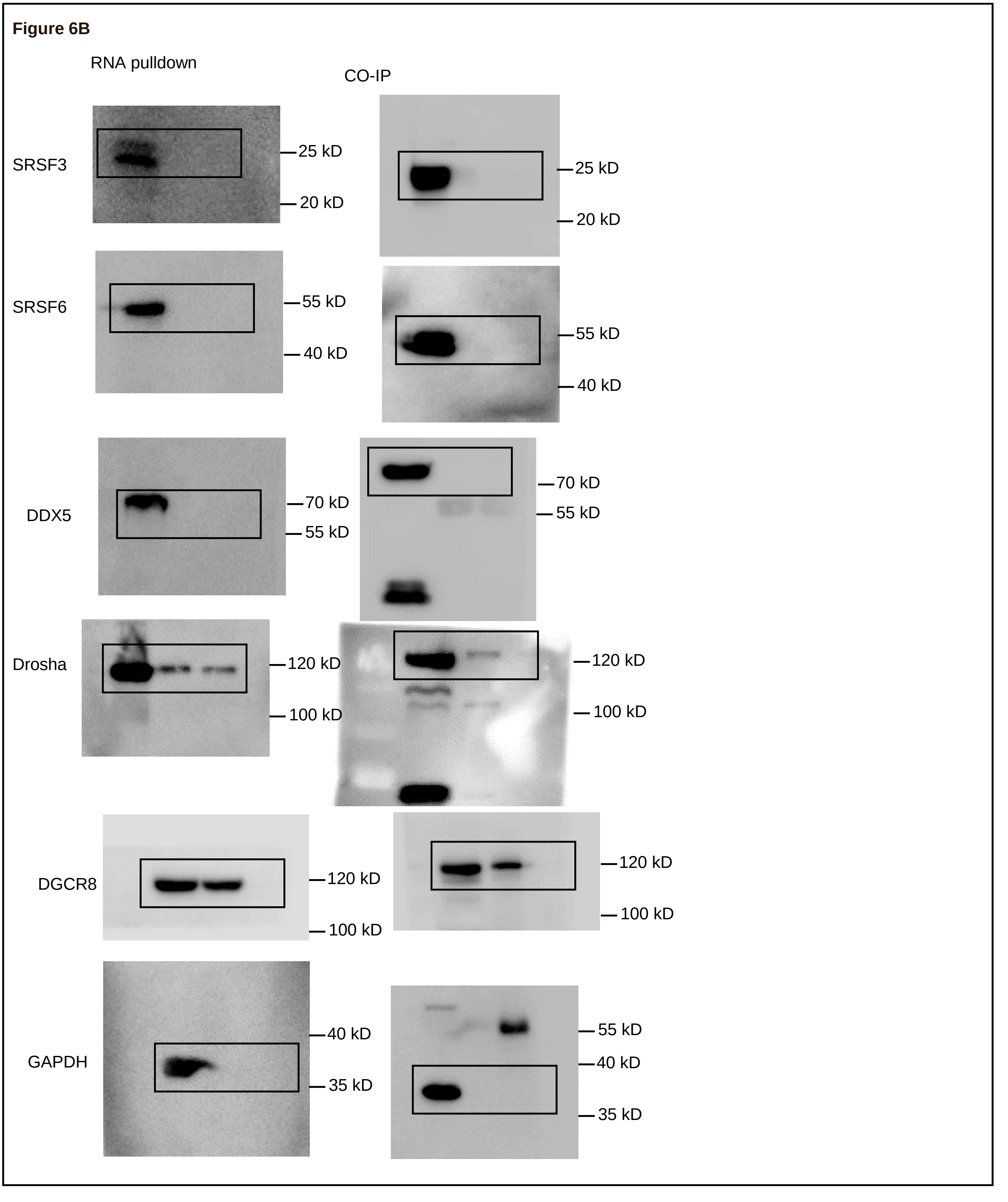

Supplementary Fig. 11. Unprocessed gel blots. (Continued)

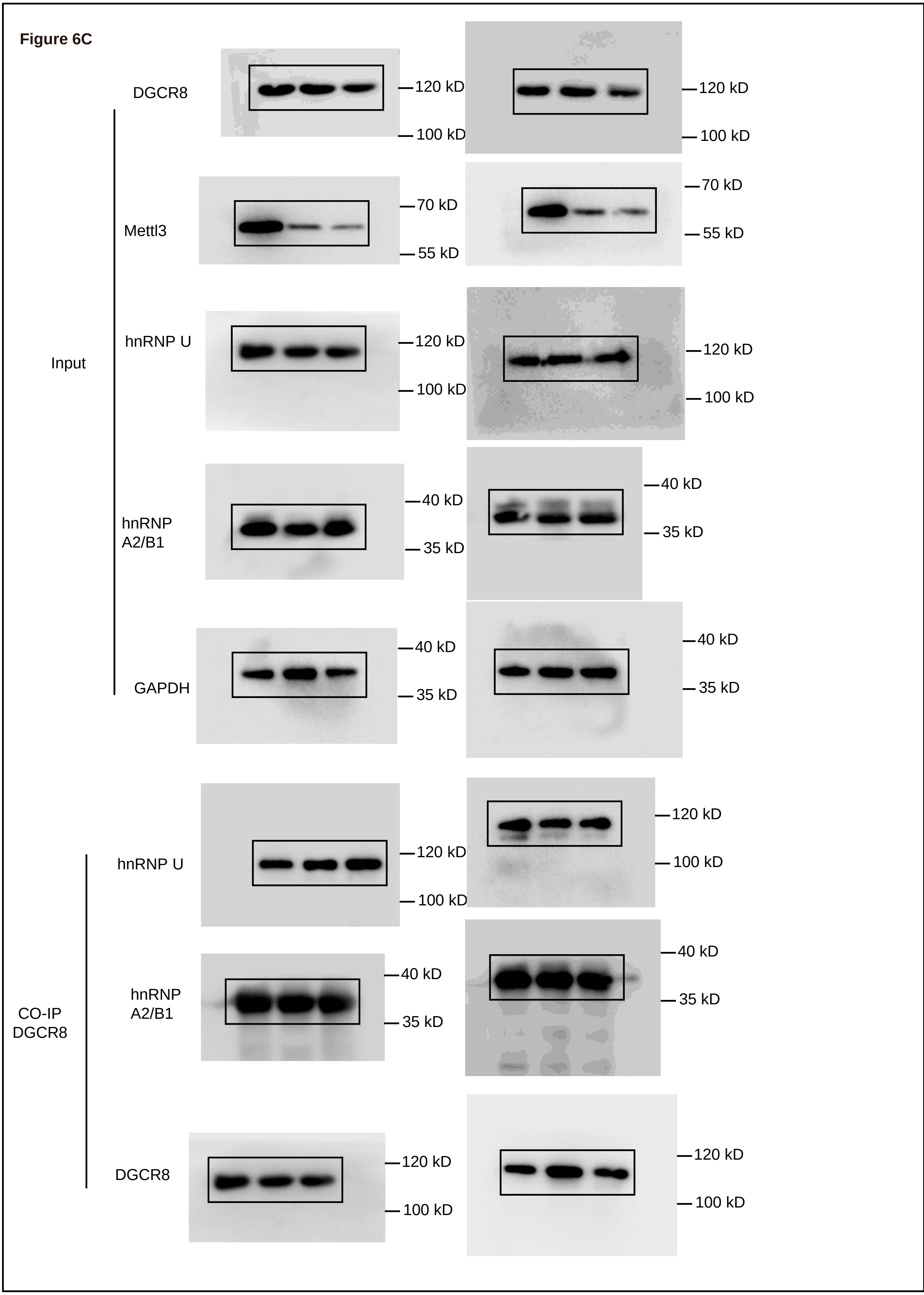

Supplementary Fig. 12. Unprocessed gel blots. (Continued)

Figure 6D

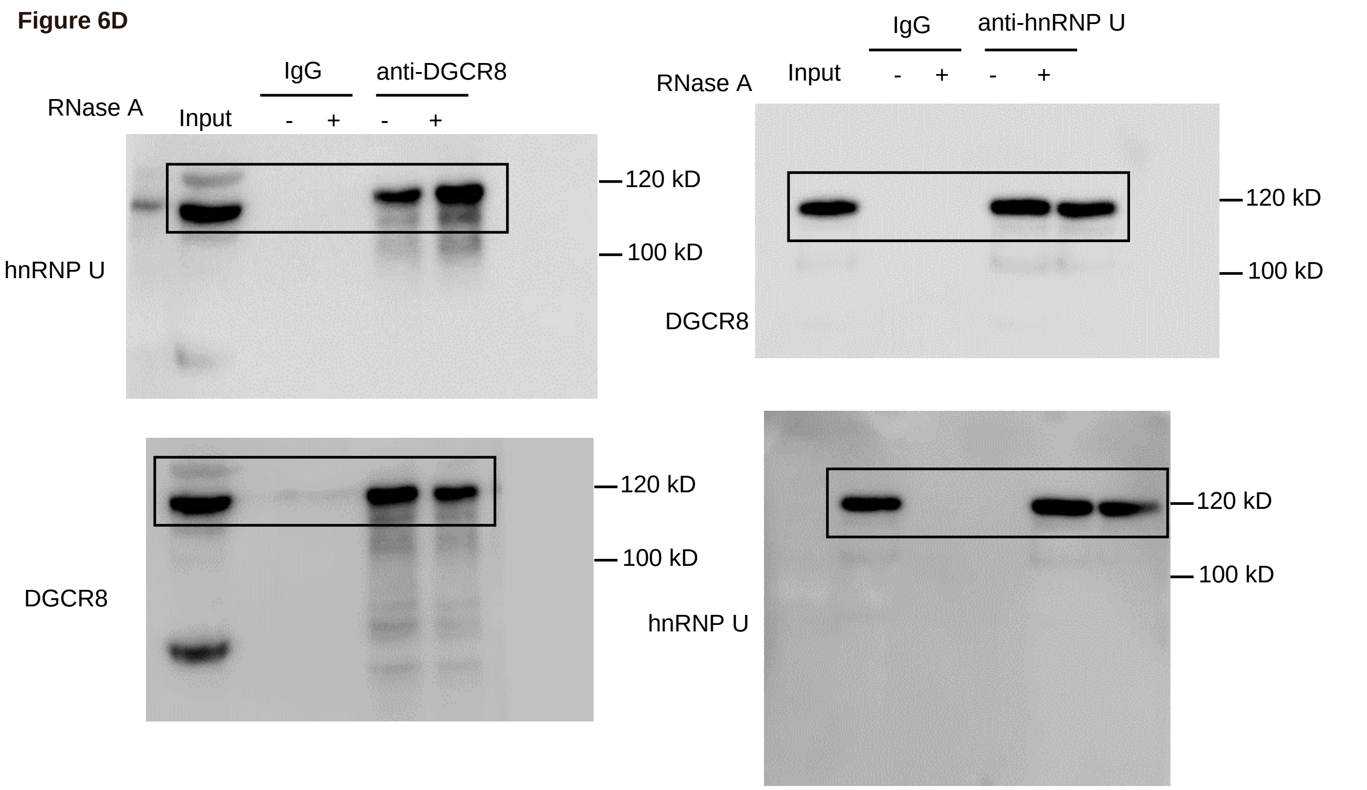

Figure 6J

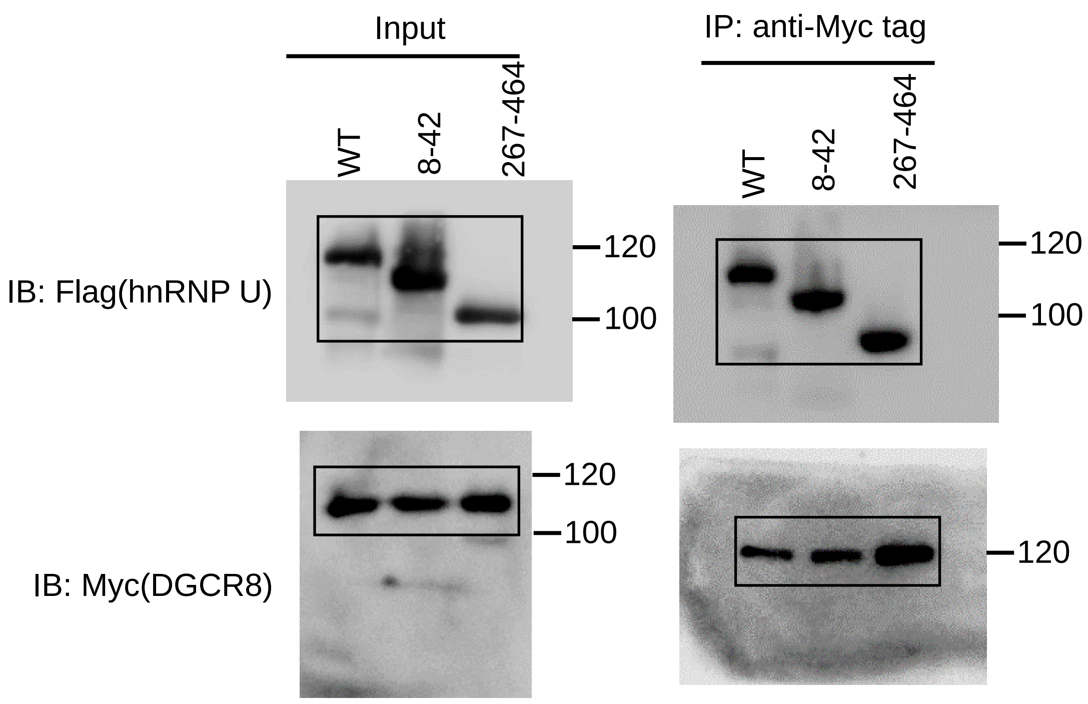

Supplementary Fig. 13. Unprocessed gel blots. (Continued)

Figure 6K

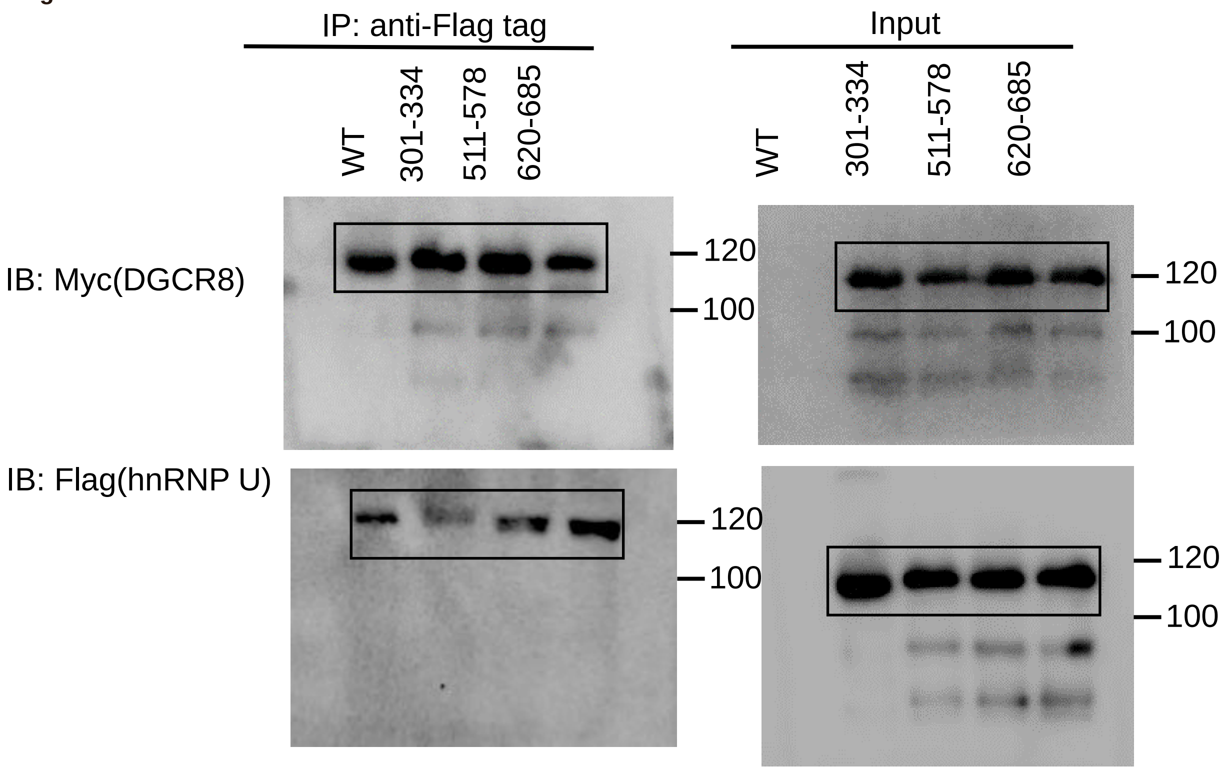

Supplementary Fig. 14. Unprocessed gel blots. (Continued)

Figure S4 A

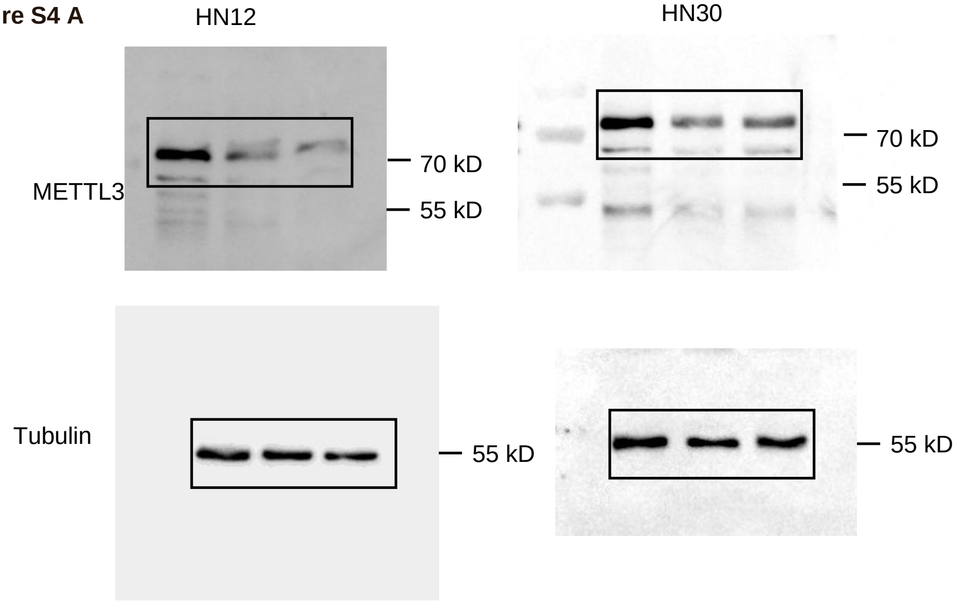

Figure S4 A

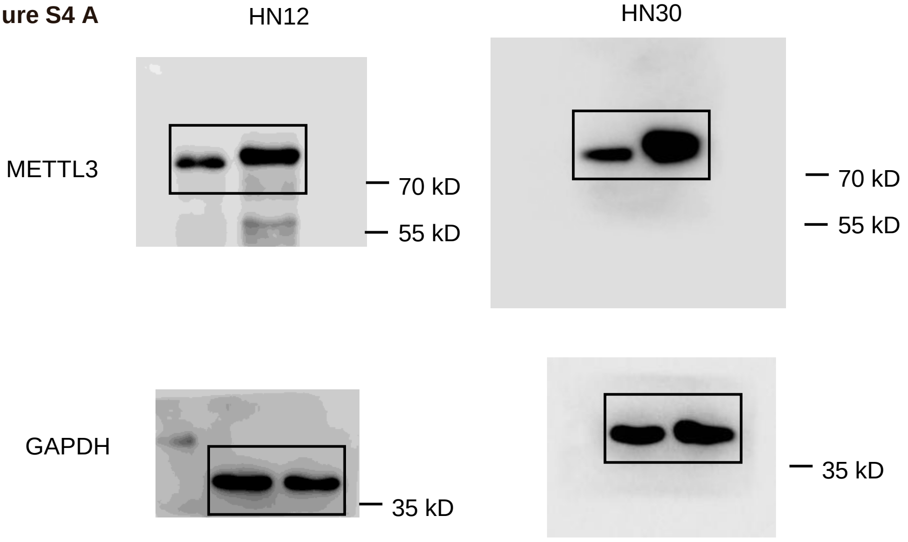

Supplement: Supplementary file 1 — Supplementary Material 1. [file 43556_2024_189_MOESM1_ESM.pdf]
